# Early contraceptive discontinuation and associated factors among married women initiating long-acting and short-acting contraceptives in humanitarian settings in Ethiopia: A retrospective cohort study

**DOI:** 10.1371/journal.pone.0345855

**Published:** 2026-03-27

**Authors:** Andamlak Gizaw Alamdo, Agnes M. Kotoh, Emefa Judith Modey, Juliana Yartey Enos

**Affiliations:** 1 Department of Health Service Management, Health Promotion, Reproductive Health, and Nutrition, School of Public Health, Saint Paul’s Hospital Millennium Medical College, Addis Ababa, Ethiopia; 2 Department of Population, Family and Reproductive Health, School of Public Health, College of Health Sciences, University of Ghana, Legon, Ghana; 3 Department of Epidemiology, Noguchi Memorial Institute for Medical Research, College of Health Sciences, University of Ghana, Legon, Ghana; University of Salamanca, SPAIN

## Abstract

**Background:**

The sexual and reproductive health (SRH) service provision in conflict-affected populations has been continually overlooked. International and local relief organizations have begun to recognize the neglect of women’s SRH needs; however, SRH services and humanitarian responses remain suboptimal. Contraceptive discontinuation is a significant public health issue with considerable impact on family planning (FP) programs, as well as on women’s SRH and population growth. There is scarce information regarding the dynamics of contraceptive use in refugee areas. Evidence on contraceptive use dynamics is vital, especially now, as the global refugee crisis grows. Therefore, this study aimed to examine first-year contraceptive discontinuation and associated factors among married women initiating long-acting reversible contraceptives (LARC) and short-acting contraceptives (SAC) in selected refugee populations in Ethiopia.

**Methods:**

A retrospective cohort study was conducted among 551 married women in selected refugee populations in Ethiopia. After reviewing FP registries, all eligible women who initiated a contraceptive method 12–18 months before data collection were included in the study. Through computer-assisted face-to-face interviews, data on the socio-demographic, reproductive, and contraceptive use history of women were collected. Key characteristics of SAC acceptors and LARC acceptors were compared using chi-square statistics and t-tests. A multivariable Cox proportional hazards model identified the predictors of time to contraceptive discontinuation during the first year of use. In addition, adjusted hazard ratios (aHR), 95% CI, and P-values were calculated to determine the magnitude and precision of the association.

**Results:**

The 12-month discontinuation rate for all contraceptive methods was 41.2% (95% CI: 37.2–45.4). The discontinuation rate for SAC (44.94%) was higher than LARC (32.32%) (P ≤ 0.001). The factors associated with 12-month discontinuation include method type (aHR = 1.50;95% CI:1.06–2.12), residence (aHR = 1.83;95% CI:1.24–2.70), husband occupation (aHR = 0.49;95% CI:0.29–0.84), length of stay in the refugee camp (aHR = 0.95;95% CI:0.92–0.99), and intention to use contraceptives in the future (aHR = 0.15;95% CI:0.11–0.21).

**Conclusion:**

A high rate of early contraceptive discontinuation was documented among refugee women, with a higher discontinuation rate among SAC acceptors. To improve contraceptive service provision and maintain continuous use of the methods initiated, targeted interventions, such as improved counselling and promotion of long-acting contraceptive methods, especially among urban refugee women, are crucial.

## Introduction

Ethiopia is the third-largest refugee-hosting nation in Africa. The country is home to over 1 million officially registered refugees and asylum seekers. South Sudan, Somalia, and Eritrea account for the majority of refugee communities [1]. Women in humanitarian settings are often unable to find contraceptive methods when only a small number would desire to become pregnant [2,3]. In addition, as a strategy for war and conflicts, women and girls are at increased risk of early and forced marriage, sexual and gender-based abuse, sexual exploitation, and transactional sex [3,4]. Therefore, refugee women critically need access to contraception for multiple reasons [4,5]. Contraception can help women plan and space their pregnancies so as not to risk having a very young baby with inadequate medical care [3]. Moreover, contraceptive provision can reduce maternal mortality and morbidity from unwanted pregnancies and unsafe abortions and help to prevent sexually transmitted infections from spreading within the population [6]. Furthermore, access to safe and reliable contraception can empower displaced women to make decisions about their bodies that are otherwise denied by society [4,7].

According to a recent systematic review and meta-analysis, the overall pooled rate of reproductive health risks among IDPs in humanitarian settings in Ethiopia was 45.8% [8]. Another study in northern Ethiopia found that 41.8% of refugee women have an unmet need for contraception. The two most common reasons for this high unmet need are the availability of contraceptive methods and women’s attitudes [9].

Contraceptive discontinuation is a significant public health issue with a considerable impact on FP programs, as well as a detrimental impact on women’s reproductive health and population growth [10]. A high rate of contraceptive discontinuation can lead to unintentional pregnancies, unintended births, and unsafe abortions, all of which raise the risk of poor newborn and child health outcomes and maternal morbidity [10,11].

Contraceptive discontinuation varies by country and method type. Evidence from 25 countries’ DHS data revealed that the contraceptive discontinuation rates of 38% and 55% were reported within the first and second year of use, respectively [6]. The discontinuation rate is higher for SAC compared to LARC, such as intrauterine devices (IUD). For instance, variation in first-year contraceptive discontinuation was reported among IUD and condom use; from 13% for those who use an intrauterine device (IUD) to 50% for condom users [12]. In Ethiopia, more than one-third (35%) of married women stopped using modern contraceptives within 3 years, and a small number (11%) of women who initiated implants discontinued their method within the first year [13].

Strong family planning services have a great impact on contraceptive continuation, method switching, and failure [14]. Evidence on the dynamics of contraceptive use helps to improve contraceptive service provision. For instance, a high rate of contraceptive discontinuation without switching to another method suggests the need to increase accessibility of different methods [10,11]. Moreover, discontinuation because of perceived side effects points to the need for better contraceptive counseling that centers on informed choice [6].

A review of the literature highlights numerous gaps in the body of knowledge on contraceptive use dynamics in humanitarian settings, and in refugee camps in particular [8,15,16]. The existing evidence mainly concentrates on contraception access and availability, and SRH challenges of women in humanitarian settings [7,15]. Although one study evaluated contraceptive discontinuation in a conflict-affected population [17], there is still a significant evidence gap on contraceptive discontinuation, method shifting, and contraceptive abandonment. Moreover, this study is in line with “the World Health Organization’s Global Research Agenda on Health, Migration, and Displacement (2023)”, which emphasises the generation of context-specific knowledge to improve access to key health services and influence policies for displaced people. Our work, which focuses on contraceptive discontinuation among refugee women in Ethiopia, contributes to this global priority and supports the WHO’s need for increased SRH research in humanitarian situations [16]. To improve equity in health outcomes and address the reproductive health needs of women in hard-to-reach situations, evidence on contraceptive discontinuation is critical, especially during the current rise of the global refugee crisis [18]. Therefore, this study aimed to examine first-year contraceptive discontinuation and associated factors among married women initiating long-acting and short-acting contraceptives in selected refugee camps in Ethiopia.

## Methods

### Study design, settings and period

A retrospective cohort study was employed to examine the time to contraceptive discontinuation and its determinants. Ethiopia offers asylum to refugee populations coming from about 19 different nations. Fifty operational partners are involved in Ethiopia’s response to the refugee crisis, including the Ethiopian government’s Refugees and Returnees Service (RRS), UNHCR (the UN Refugee Agency), and international and national NGOs. As of December 31, 2023, Ethiopia was the third-largest refugee-hosting nation in Africa, with 1,043,602 officially registered refugees and asylum seekers. Of these, males account for 48%, while females account for 52%. South Sudan and Somalia account for the majority of the refugee communities [1]. This study was conducted among Somali refugees in the Kebribeyah, Sheder, and Awubare refugee camps, and South Sudanese refugees in the Jewi refugee camp in Ethiopia. According to the UNHCR report for 2023, the total population of Kebribeyah, Sheder, Awubare, and Jewi refugee camps is 18,043, 14,695, 13,413, and 67,896, respectively. In terms of administrative location, the Kebribeyah and Awubare refugee camps are urban, but Sheder and Jewi are rural. Furthermore, each refugee camp has a health centre (RRS HC) which provides health care services, including FP [19].

Somali and South Sudanese refugee camps found in Gambella and the Somali region were selected due to the large number of refugee populations. In addition to the accessibility, safety, and security to travel to the study participants’ residential areas, refugee communities from Sudan, Eritrea, and Yemen were not included in the study due to the small number of refugee populations and women in particular. The study was conducted from March 1 to April 30, 2024.

### Study participants and selection procedure

The majority of FP clients at the RRS health center in selected refugee camps were married women, and the health facility registries primarily document information for married women. Furthermore, the sociocultural norms in these refugee camps often associate the use of contraceptives with marriage.

Thus, married women in refugee camps who initiated SAC or LARC 12–18 months before data collection began were identified from the FP registry books of the health facilities and included in the study. Users whose timing of contraceptive initiation was not documented, married women whose follow-up records were incomplete, and those women with hearing impairment were excluded from the study.

### Sample size determination

In the calculation of the sample size for time to contraceptive discontinuation, survival analysis was considered based on the following underlying assumptions: two population proportions (q0 = proportion of the unexposed group and q1 = proportion of the exposed group), the statistical power of 80%, 95% CI, and one-year discontinuation probability and hazard ratio (HR) from a previous similar study conducted in the conflict-affected population in Congo [17]. We considered women who utilized SAC as an exposure variable and LARC as unexposed.

Where:

q0 = proportion of LARC users = 55.47%

q1 = proportion of SAC users = 44.52%

HR = 1.74 (a reported adjusted HR of 1.74 for discontinuation-related with the use of SAC Vs LARC in conflict-affected settings in Congo)

The probability of one-year discontinuation(E) = 18.4% (Casey et al., 2017) [17].

Alpha (two-sided); 0.05; Zα/2 = 1.96

Power = 80%; Zβ = 0.841

Number of events needed (E)


$$E=\frac{(\text{Z}\alpha /\text{2}+\text{Z}\beta {)}^{2}}{(\text{Log HR}{)}^{2}*\text{q0}*\text{q1}}=\text{104}$$

Sample size (n)


$$n=\frac{E}{P(E)}=\frac{104}{18.4}\%=566$$

In practice, we have included all eligible women who have complete records (551 participants) in selected refugee camps (LARC = 164 and SAC = 387) between September 2022 and February 2023. This strategy enabled us to capture the all accessible population while maximising representativeness.

### Sampling technique

**Fig 1:**
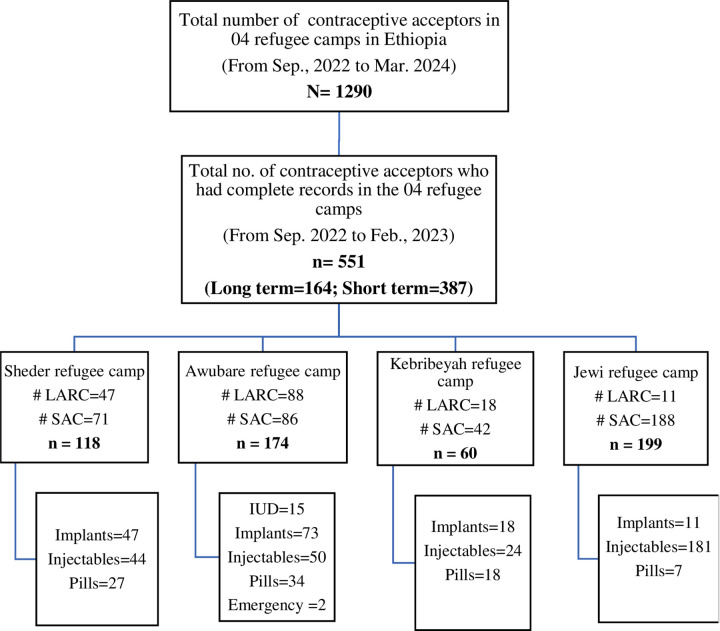
Schematic presentation of the distribution of contraceptive initiations in Sheder, Awubare, Kebribeyah, and Jewi refugee camps in Ethiopia, 2024.

First, the Gambella and Somali regions, which have the highest refugee populations in Ethiopia, were selected. Next, three refugee camps from the Somali region and two from the Gambella region were chosen using a lottery method from a total of 14 refugee camps (8 Somali and 6 South Sudanese). Later, we excluded one refugee camp from the Gambella region, due to safety and security concerns when traveling to the study participants’ residential areas. The excluded camp had refugees with similar characteristics to those included. Each refugee camp has one health center that provides health care services, including family planning. Therefore, 4 health centers were visited, and a total of 1290 women who initiated SAC or LARC 12–18 months before data collection were identified from the registers of health facilities. To have a follow-up period of at least one year, only those refugee women who had initiated contraceptive methods between September 01, 2022, and February 30, 2023, were selected from the list of women who had complete records in all refugee camps. Accordingly, only 551 refugee women who started LARC (164) and SAC (387) (between September 2022 and February 2023) had complete records and were eligible to participate in the study. Eight women were excluded: one had hearing issues, one was ill at the time of the interview, and six had incomplete records. Because the total number of eligible refugee women was comparable with the sample size calculated (566) to maintain optimal statistical power, all 551 samples were included in the study (Fig 1). Since the rate of contraceptive discontinuation among SAC and LARC acceptors is assumed to differ, the two groups were treated differently. The study participants were located in collaboration with health workers and community health agents, and interviews were conducted in the women’s homes. Only health professionals and community health agents who were already familiar with the women and offered services at healthcare facilities made the initial contact with potential participants.

### Data collection tool and procedure

First, FP registration books, follow-up registration forms, and contraception reporting forms in the health facilities (in the immediate past 18 months) were reviewed. Data were collected using computer-assisted face-to-face interviews using the Open Data Kit (ODK). The tool was initially developed in English and translated into the local languages (Somali and Nuer), and then, to ensure consistency and correctness, the language experts back-translated the tool into English. The questionnaire was developed based on the review of relevant literature [14,17,20]. By using face-to-face interview techniques, relevant data on the socio-demographic (age, country of origin, husband occupation, etc.), reproductive (pregnancy status, number of living children, history of abortion, etc.), and contraceptive use history of women (sources of information, type of method, etc.) were collected (S1 File). Ten data collectors with a BSc degree in public health and midwifery and 4 supervisors with master’s degrees in public health were recruited and trained before they were deployed for data collection. Considering the country’s current security issues, the data collection was undertaken while closely following the local security situation.

### Study Variables

The dependent variable was the time to contraceptive discontinuation during the first year of use among women initiating short—and long-acting reversible contraceptives in humanitarian settings in Ethiopia. Independent variables include socio-demographic characteristics such as religion, age, educational status, country of origin; husband’s level of education, occupation, birth, and reproductive histories such as gravidity, number of children, and contraceptive method used. Related variables include the type of contraceptives and side effects, etc.

- Contraceptive discontinuation: Contraceptive discontinuation was assessed only among women who initiated contraceptive use 12–18 months before data collection. A woman was defined as a “continuer” if she was using the same method until the data collection period; she was defined as a “switcher” if she was using a different method than what she had been using 12–18 months before the data collection; and she was defined as a “discontinuer” if she reported non-use during the interview [14,17].
- Abandonment: Termination of using any method altogether or discontinuation that is not followed by the restart of use at post-discontinuation [14,17].
- Method failure: Method failure was captured by this study as the identification of a pregnancy that was reported to have occurred alongside the participant’s use of a contraceptive method, but not reported as “wanted to become pregnant” [14,17].

### Data quality control

Data collection was carried out using the electronic method Open Data Kit (ODK). This approach enables a paperless and more precise data collection process, saving time that would otherwise be spent on data entry. Additionally, the questionnaire was pretested for language clarity and response suitability. The pre-test involved 5% of the total sample of women (28 women) who started contraceptive methods outside the study area. Moreover, the research team received extensive training before the actual data collection, including practical field exercises, especially focusing on the use of electronic data collection.

### Data processing and analysis

Before data analysis, data cleaning was carried out through a series of cross-tabulations, frequency tables, and raw data checkups. Stata16 (StataCorp, College Station, Texas, USA), was used for all data analysis. For each question, descriptive statistics such as frequency, percentage, means, and standard deviations were used to explain the different socio-demographics, birth and reproductive history, and contraceptive use information of the women by the baseline contraceptive type. A log-rank test was used to select potential candidate categorical predictors of contraceptive discontinuation within 12 months of use. Kaplan-Meier survival estimates were plotted to test the significance of observed differences between the baseline contraceptive types.

A multivariable Cox proportional hazards model identified predictors of the time to contraceptive discontinuation during the first year of use. Adjusted hazard ratios (AHR), 95% CI, and P-values were calculated to assess the statistical significance and strength of the association between the factors and the dependent variable. Covariate selection for the Cox proportional hazards model was guided by conceptual relevance, evidence from prior literature, and statistical screening. A final Cox proportional hazards model was fitted by including all potential predictors with a significance level of <0.25 in the log-rank test, along with variables that showed statistical significance with the dependent variables in other studies. All possible interactions were evaluated, and those that were statistically significant were retained for further analysis. The proportional hazards assumptions were checked using Schoenfeld residuals. Both global and covariate-specific tests were conducted. Covariates with p-values > 0.05 were considered to satisfy the PH assumption. The Akaike Information Criterion (AIC) was used to guide model selection, with the lowest AIC indicating the best-fitting model while considering model complexity. A P-value less than 0.05 was deemed statistically significant. Finally, the study’s findings were presented using narrations, tables, and charts.

### Ethical considerations

First, ethical approval was obtained from the IRB of St. Paul’s Hospital Millennium Medical College (SPHMMC) Research and Resources Mobilization Directorate (Ref. No: PM23/401/23/01/2024). Additionally, permission and support letters were secured from all participating institutions, including the Federal Democratic Republic of Ethiopia Refugees and Returnees Service (RRS), Gambella Region RRS (Gambella branch office), and the Somali Region RRS (Jigjiga branch office). Most study participants used contraceptive methods without the knowledge of their male partners and mothers-in-law. Asking for written informed consent could cause discomfort for the participants. Furthermore, the SPHMMC IRB generally requires verbal informed consent for all research other than clinical trials involving invasive procedures. Health providers and community health agents who were already familiar with the refugee women initially contacted and introduced the purpose of the study. Then, data collectors obtained verbal informed consent after discussing the study objectives, procedures, requirements, potential benefits and risks, privacy, and data confidentiality with participants using simple language. After reading the participant’s consent statement, the interviewer signed a record confirming that consent was obtained. All participants were informed that they could withdraw their consent at any time without penalty. Additionally, privacy and confidentiality were maintained by not exposing individual identifiers, not sharing data with third parties, and using it solely for research purposes.

### Inclusivity in global research

Additional information regarding the ethical, cultural, and scientific considerations specific to inclusivity in global research is included in the Supporting Information (S1 Checklist)

## Results

A total of 1290 refugee women initiated long and short-acting contraceptive methods between September 2022 and February 2024 in health facilities providing healthcare services for the refugee population. Of these, 551 refugee women who initiated long-acting (164) or short-acting (387) contraceptive methods within 12–18 months before the date of data collection were eligible and included in the study. Over twenty-one percent (21.4%) of contraceptive acceptors were selected from Sheder, 31.6% from Awubare, 10.9% from Kebribeyah, and 36.1% from Jewi refugee camp (Fig 1).

### Socio-demographic and background information

Table 1 presents the respondents’ socio-demographic and background characteristics by baseline contraceptive type. Overall, the mean age at the time of contraceptive initiation (first time use of a method) was 26.6 (±5.8) while the mean age of the respondents (current age) was 29.9 (±6.5). Both the mean age (P = 0.165) and the mean age at the time of contraceptive initiation (P ≤ 0.615) were not significantly different for LARC and SAC acceptors. Nearly all (93.3%) LARC and 51.4% of SAC acceptors were displaced from Somalia (P ≤ 0.001), and they were Muslim by religion. Moreover, the mean length of stay in refugee camps for LARC acceptors was 14.7 (±4.1) years while it was 12.0 (±5.2) years for SAC acceptors (P ≤ 0.001). Over two-thirds of SAC (68.5%) and 59.2% of LARC acceptors ever attended school (P = 0.035). More than one-third of LARC (35.4%) and 66.4% of SAC acceptors were residing in the rural refugee camps (P ≤ 0.001). Concerning the occupation of the respondents, 78.1% of LARC and 76.7% of SAC acceptors were housewives/not working (P = 0.815). Regarding the self-declared wealth, 42.1% of LARC and 40.1% of SAC acceptors belonged to the poorer self-declared wealth quintile (P = 0.015). Furthermore, over a third of LARC (35.4%) and 37.7% of SAC acceptors’ husbands had no formal education (P ≤ 0.001), while 23.2% and 42.6% of SAC acceptors’ husbands were not working (P ≤ 0.001) (Table 1).

**Table 1:** Socio-demographic and background information of the respondents by contraceptive method type in humanitarian settings, Ethiopia, 2024 (n = 551).

Variables/Category		Total contraceptive users (551(%))	LARC acceptors (164(%))	SAC acceptors (387(%))	P-value
Mean age at the time of contraceptive initiation (SD)		26.6(5.8)	26.8(5.5)	26.5(6.0)	0.615
Mean age (SD)		29.93(6.5)	30.53(6.1)	29.7(6.7)	0.165
Age groups	15-24	116(21.1)	22(13.4)	94(24.3)	0.016*
	25-34	305(55.4)	101(61.6)	204(52.7)	
	35-49	130(23.6)	41(25.0)	89(23.0)	
Country of origin	Somalia	352(63.9)	153(93.3)	199(51.4)	P < 0.001*
	South Sudan	199(36.1)	11(6.7)	188(48.6)	
Ever attended school	No	189(34.3)	67(40.9)	122(31.5)	0.035*
	Yes	362(65.7)	97(59.2)	265(68.5)	
Educational level (n = 362)	No formal education	69(19.1)	21(21.7)	48(18.1)	P < 0.001*
	Primary	138(38.1)	21(21.7)	117(44.2)	
	Secondary	115(31.8)	28(28.9)	87(32.8)	
	Higher	40(11.1)	27(27.8)	13(4.9)	
Religion	Muslim	352(63.9)	153(93.3)	199(51.4)	P < 0.001*
	Protestant	165(29.9)	11(6.7)	154(39.8)	
	Adventist	18(3.3)	0(0.0)	18(4.7)	
	Other Christian	16(2.9)	0(0.0)	16(4.1)	
Residence	Rural	315(57.2)	58(35.4)	257(66.4)	P < 0.001*
	Urban	236(42.8)	106(64.6)	130(33.6)	
Occupation	Housewife/Not working	425(77.1)	128(78.1)	297(76.7)	0.815
	Merchant	95(17.2)	27(16.7)	68(17.6)	
	Private employee	24(4.4)	6(3.7)	18(4.7)	
	Other^a^	7(1.3)	3(1.8)	4(1.0)	
Self-declared wealth	Poorest	159(28.9)	33(20.1)	126(32.6)	0.015*
	Poorer	224(40.7)	69(42.1)	155(40.1)	
	Middle	150(27.2)	55(33.5)	95(24.6)	
	Richer	18(3.3)	7(4.3)	11(2.8)	
Husband education	No formal education	204(37.0)	58(35.4)	146(37.7)	0.010*
	Primary	119(21.6)	27(16.5)	92(23.8)	
	Secondary	147(26.7)	43(26.2)	104(26.9)	
	Higher	81(14.7)	36(22.0)	45(11.6)	
Husband occupation	Notworking	203(36.8)	38(23.2)	165(42.6)	P < 0.001*
	Merchant	138(25.1)	50(30.5)	88(22.7)	
	Day labor	136(24.7)	46(28.1)	90(23.3)	
	Private employee	62(11.3)	25(15.2)	37(9.6)	
	Other^b^	12(2.2)	5(3.1)	7(1.8)	
Mean length of stay in refugee camp in years (SD)		12.8(5.1)	14.7(4.1)	12.0(5.2)	P < 0.001*

* Represents statistically significant association; SD: Standard Deviation.

*LARC: Long-acting reversible contraceptive methods; SAC: Short-acting contraceptive methods.*

*Other Christian: Orthodox, catholic,etc.; Other* ^*a:*^ *: Government employee, charcoal maker, etc.*

*Other* ^*b*^ *: Farmer, Government employee, etc.*

### Birth and reproductive health information of the respondents

Almost all LARC (98.8%) and 92.8% of SAC acceptors had ever been pregnant (P = 0.004). The overall mean gravidity and the mean number of children for respondents in this study were 4.2 (±2.5) and 3.7 (±2.2), respectively. The study revealed higher mean gravidity and mean number of children for LARC acceptors compared to SAC acceptors (P ≤ 0.001). Similarly, there was a significant difference in the history of miscarriage (P = 0.025) and plan to get pregnant for the recent pregnancy (P ≤ 0.001) among the LARC and SAC acceptors. Among respondents who had ever been pregnant, 10.2% were pregnant during the time of data collection, 6.3% had a history of abortion, 83.5% had ANC follow-up for the most recent pregnancy, and 16.5% had a desire for more children within 2 years (Table 2).

**Table 2:** Reproductive health information of the respondents by contraceptive method type in humanitarian settings, Ethiopia, 2024 (n = 551).

Variables/Category		Total contraceptive users (551(%))	LARC acceptors (164(%))	SAC acceptors (387(%))	P-value
Mean gravidity (SD)		4.2(2.5)	5.2(2.6)	3.9(3.4)	P < 0.001*
Mean number of children (SD)		3.7(2.2)	4.5(2.1)	3.4(2.1)	P < 0.001*
Ever been pregnant	Yes	521(94.6)	162(98.8)	359(92.8)	0.004*
Currently pregnant (n = 521)	Yes	56(10.2)	13(7.9)	43(11.1)	0.258
Plan to get pregnant for the recent pregnancy (n = 521)	Then	331(63.5)	122(75.3)	209(58.2)	P < 0.001*
	Later	178(34.2)	37(22.8)	141(39.3)	
	Not at all	12(2.3)	3(1.9)	9(2.5)	
Ever had miscarriage (n = 521)	Yes	140(26.9)	54(33.3)	86(24.0)	0.025*
Ever had abortion (n = 521)	Yes	33(6.3)	10(6.2)	23(6.4)	0.919
ANC for the recent pregnancy (n = 521)	Yes	435(83.5)	138(85.2)	297(82.7)	0.485
Desire for more children	Within 2 years	91(16.5)	21(12.8)	70(18.1)	0.246
	After 2 years	421(76.4)	129(78.7)	292(75.5)	
	Wants no more children	39(7.1)	14(8.5)	25(6.5)	

* Represents statistically significant association; SD: Standard Deviation

*NB: desire for more children assessed relative to the time of interview/now*

### Contraceptive use-related information of the respondents

Compared to women who initiated SAC (42.6%), a higher proportion of LARC acceptors (57.3%) had a history of contraceptive use before the baseline method (P = 0.002). In addition, 67.7% LARC and 42.1% of SAC acceptors’ husbands were aware of their wives’ use of contraceptive methods (P ≤ 0.001). Moreover, partner approval of contraceptive use was higher among LARC acceptors compared to SAC acceptors (P ≤ 0.001). Concerning the decision to start contraception, 73.4% of SAC and 56.1% of LARC acceptors decided by themselves (P ≤ 0.001). There was no significant difference in the provision of counseling service before initiation of the baseline method (P = 0.321) and intention to use contraception in the future (P = 0.908) among LARC and SAC acceptors (Table 3).

**Table 3:** Contraceptive use information of the respondents by contraceptive method type in humanitarian settings, Ethiopia, 2024 (n = 551).

Variables/Category		Total contraceptive users (551(%))	LARC acceptors (164(%))	SAC acceptors (387(%))	P-value
Contraceptive use before the baseline method	Yes	259(47.0)	94(57.3)	165(42.6)	0.002*
Husband aware of contraceptive use	Yes	274(49.7)	111(67.7)	163(42.1)	P < 0.001*
Husband approves contraceptive use	Yes	245(44.5)	108(65.9)	137(35.4)	P < 0.001*
Decision to start contraception	Mainly woman	376(68.2)	92(56.1)	284(73.4)	P < 0.001*
	Jointly	122(22.1)	63(38.4)	59(15.3)	
	Health professional	46(8.4)	9(5.5)	37(9.6)	
	Mainly husband	7(1.3)	0(0.0)	7(1.8)	
Got counseling services	No	20(3.6)	8(4.9)	12(3.1)	0.321
	Yes	525(95.3)	153(93.3)	372(96.1)	
	I don’t remember	6(1.1)	3(1.8)	3(0.8)	
Intention to use contraception in the future	Yes	405(73.5)	120(73.2)	285(73.6)	0.908

* Represents statistically significant association

### Contraceptive use dynamics among the respondents

**Fig 2:**
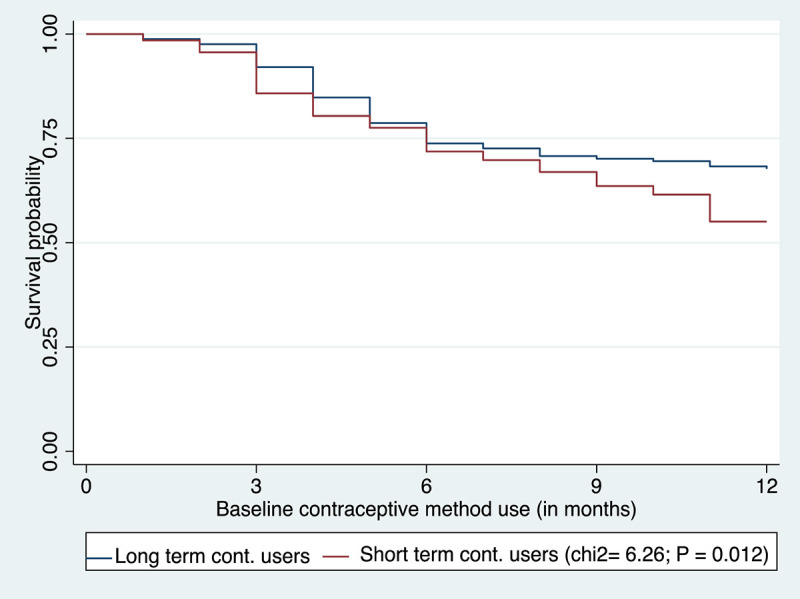
Kaplan-Meier survival curve for contraceptive discontinuation among LARC and SAC acceptors in humanitarian settings Ethiopia, 2024.

**Fig 3:**
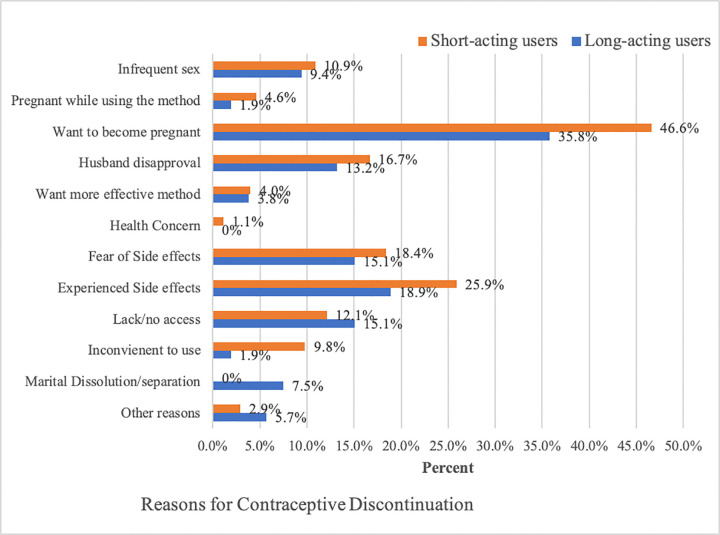
Reasons for contraceptive discontinuation among LARC and SAC acceptors in humanitarian settings Ethiopia, 2024.

At 12 months, 41.2% (95% CI: 37.2–45.4) of women discontinued their baseline contraceptive methods. The contraceptive discontinuation rate was 12.3% (95% CI: 9.8–15.3) at three months and 27.5% (95% CI: 24.0–31.5) at six months. A higher proportion of discontinuation among SAC acceptors (45.0%) was observed compared to LARC acceptors (32.3%) (P ≤ 0.001). Specifically, the discontinuation for SAC acceptors (emergency contraceptive pills 2 (2), oral contraceptive pills 43 (50%), and injectables 129 (43.1%)) and for LARC acceptors (IUD 6 (40%) and implants 47 (31.5%)). Fig 2 below shows the Kaplan-Meier survival estimates for the time to discontinuation of a method among LARC and SAC acceptors (P = 0.012). This illustrates a significantly lower rate of contraceptive discontinuation among LARC acceptors compared to women who initiated SAC. The main reasons given for contraceptive discontinuation include wanting to become pregnant (LARC (35.8%) vs. SAC (46.6%)), experiencing side effects (LARC (18.9%) vs. SAC (25.9%)), fear of side effects (LARC (15.1%) vs. SAC (18.4%)), husband disapproval (LARC (13.2%) vs. SAC (16.7%)), and lack of/no access to the services (LARC (15.1%) vs. SAC (9.8%)) (Fig 3). Fifteen percent of LARC users and 12.1% of SAC users reported lack of or limited access to services as a reason for discontinuation. As multiple responses were permitted, these percentages reflect participants who cited lack of access as one of several reasons, rather than necessarily the primary reason. This finding should be interpreted in the context of refugee women, for whom family planning services, particularly LARC, may be inconsistently available, making follow-up care or timely removal challenging. There was no significant difference in the method switch to a modern method (P = 0.254) and the time for the method switch among the LARC and SAC acceptors (P = 0.440). Among women who discontinued the baseline methods, 60.4% of LARC and 32.18% of SAC acceptors abandoned the method. Regarding the contraceptive method failure, 8.5% of women reported a method failure in their lifetime. Of these, 14.9% (n = 7) reported method failure while using implants, and 42.6% of women (n = 20) reported method failures while using injectables and oral contraceptive pills (Table 4).

**Table 4:** Contraceptive use dynamics (contraceptive discontinuation, method switch, contraceptive abandonment, and method failure) among respondents who initiated the baseline contraceptive methods in humanitarian settings, Ethiopia, 2024 (n = 551).

Variables/Category		Total contraceptive users (551(%))	LARC acceptors (n = 164(%))	SAC acceptors (387(%))	P-value
Discontinuation of the baseline method	Yes	227(41.2)	53(32.3)	174(45.0)	0.006*
Method switch (N = 227)	Yes	32(14.1)	10(18.9)	22(12.6)	0.254
Time for method switch (n = 32)	In the same month	10(31.3)	2(20.0)	8(36.4)	0.440
	After 1 month	22(68.7)	8(80.0)	14(63.6)	
Contraceptive abandonment (n = 227)	Yes	88(38.8)	32(60.4)	56(32.2)	P < 0.001*
Ever had method failure	Yes	47(8.5)	16(9.8)	31(8.0)	0.502
Method failure while using which method? (n = 47)	Implants	7(14.9)	7(43.8)	0(0.0)	P < 0.001*
	Injectables	20(42.6)	3(18.6)	17(54.8)	
	Contraceptive pills	20(42.6)	6(37.5)	14(45.2)	

* Represents statistically significant association

### Determinants of early contraceptive discontinuation

The bivariate analysis revealed twelve potential predictors of early contraceptive discontinuation, including variables with a significance level below 0.25 in the log-rank test.

The final Cox proportional hazards model demonstrated good fit to the data (likelihood ratio χ²(21) = 235.52, p < 0.001) with an Akaike Information Criterion (AIC) of 2575.92, indicating adequate model performance.

Of the potential predictors, contraceptive method type, residence, length of stay in the refugee camp, husband’s occupation, and intention to use contraception showed significant association with the hazard of contraceptive discontinuation in the final Cox regression model. Specifically, the risk of contraceptive discontinuation within 12 months of use among SAC acceptors was 50% higher than LARC acceptors (aHR = 1.50; 95% CI: 1.06–2.12). The likelihood of contraceptive discontinuation during the first year of use for a refugee woman residing in an urban area was 83% higher than that of a woman from a rural area (aHR = 1.83; 95% CI: 1.24–2.70). Concerning the husband’s occupation, women whose husbands were employed in the private sector had a 51% reduced risk of contraceptive discontinuation within 12 months of use compared to a husband with a non-working occupational status (aHR = 0.49; 95% CI: 0.29–0.84). Additionally, the study revealed that a one-year increase in the length of stay in the refugee camp for a refugee woman results in a 5% lower risk of contraceptive discontinuation (aHR = 0.95; 95% CI: 0.92–0.99). Furthermore, refugee women who had an intention to use contraception in the future had an 85% lower risk of contraceptive discontinuation within the first year of use (aHR = 0.15; 95% CI: 0.11–0.21) (Table 5).

**Table 5:** Multivariable Cox regression analysis of determinants of contraceptive discontinuation within the first year of use among married women in humanitarian settings, Ethiopia, 2024.

Variables/Category		cHR(95% CI)	aHR(95% CI)	P-value
Contraceptive method type	Long acting	1(ref)	1(ref)	
	Short acting	1.46(1.07-1.98)	1.50(1.06-2.12)	0.022*
Age at the time of contraceptive initiation	15-24	1(ref)	1(ref)	
	25-34	0.77(0.58-1.03)	0.83(0.59-1.16)	0.282
	35-49	1.48(1.01-2.16)	0.97(0.62-1.52)	0.909
Residence	Rural	1(ref)	1(ref)	
	Urban	1.69(1.30-2.19)	1.83(1.24-2.70)	0.002**
Self-declared wealth	Poorest	1(ref)	1(ref)	
	Poorer	1.04(0.73-1.47)	1.23(0.84-1.79)	0.278
	Middle	1.91(1.36-2.69)	1.46(0.94-2.27)	0.091
	Richer	2.00(1.01-3.93)	1.89(0.88-4.02)	0.098
Husband occupation	Not working	1(ref)	1(ref)	
	Merchant	1.23(0.88-1.72)	0.71(0.47-1.08)	0.115
	Day labor	1.21(0.86-1.71)	0.95(0.62-1.45)	0.831
	Private employee	0.87(0.53-1.41)	0.49(0.29-0.84)	0.010*
	Other^b^	2.55(1.31-4.93)	1.15(0.56-2.37)	0.690
Length of stay in refugee camp in year		0.97(0.94-1.00))	0.95(0.92-0.99)	0.020*
No, of children	0-1	1(ref)	1(ref)	
	2-4	0.62(0.43-0.89)	0.83(0.55-1.23)	0.365
	5-7	0.72(0.49-1.06)	0.91(0.55-1.52	0.743
	8+	1.40(0.75-2.60)	1.72(0.80-3.66)	0.160
Desire for more children	Within 2 years	1(ref)	1(ref)	
	After 2 years or wants no more children	0.54(0.40-0.93)	0.98(0.68-1.40)	0.918
Contraceptive use before the baseline method	No	1(ref)	1(ref)	
	Yes	1.04(0.80-1.36)	0.82(0.59-1.13)	0.23
Husband approves the contraceptive use	No	1(ref)	1(ref)	
	Yes	0.58(0.44-0.76)	0.91(0.67-1.25)	0.60
Got counseling services	No	1(ref)	1(ref)	
	Yes	1.27(0.60-2.71)	1.41(0.65-3.06)	0.381
	I don’t remember	0.43(0.53-3.53)	1.12(0.13-9.49)	0.91
Intention to use contraception in the future	No	1(ref)	1(ref)	
	Yes	0.14(0.11-0.19)	0.15(0.11-0.21)	0.001**

*cHR: crude hazard ratio; aHR; adjusted hazard ratio;*

** Represents statistically significant association (p < 0.01)

* Represents statistically significant association (p < 0.05)

*1 (ref): Indicates the reference/the baseline category*

*Other* ^*b*^ *: Farmer, government employee, etc.*

## Discussion

Our study reported the first-year contraceptive discontinuation rate and related factors among married women starting long-acting and short-acting contraceptives in selected refugee populations in Ethiopia. Forty-one percent of women discontinued their initial contraceptive method within 12 months, with a higher discontinuation rate among SAC acceptors compared to LARC acceptors. This rate was higher than those found in studies from Uganda and Congo [17,20]. In Uganda, a lower overall discontinuation rate of 6.8% was reported based on secondary data [20]. Additionally, among the conflict-affected population in Congo, the 12-month contraceptive discontinuation rate was only 18.4% [17]. A large DHS calendar data analysis across 61 countries found an overall discontinuation rate of 10.5%. However, results varied based on user characteristics, country, and method type. For example, the study showed that the discontinuation rates for OCP and injectables in SSA were 23.4 and 23.5%, respectively [21].

Compared to the general population of Ethiopia, we found higher contraceptive discontinuation rates in our study population [22]. More than a quarter of (27.1%) Ethiopian women who started using modern contraceptive methods discontinued the methods prematurely [23]. Similarly, a contraceptive discontinuation rate of 32.2%, for all modern contraceptive methods, was reported in a community-based study in Ethiopia [10]. Moreover, a meta-analysis of LARC use in Ethiopia revealed a pooled discontinuation rate of 36.9% [24]. Our findings are comparable to a 1-year overall contraceptive discontinuation rate (39%) reported in a study conducted in Myanmar, which found a higher discontinuation rate from the SAC users, particularly pills (43%) and injectables (42%), and very low contraceptive discontinuation for LARC such as IUDs (7%) and contraceptive implants (0.2%) [25]. Furthermore, a similar report by Ross indicated a discontinuation rate of over 40% among reproductive-age women in developing countries for SAC at 12 months in 2017 [26].

The higher first-year contraceptive discontinuation rate in our study could be related to differences in the study population, method mix, recall period, study settings, and access to healthcare services. Specifically, contraceptive discontinuation in refugee women could be higher for multiple reasons. Among others, refugee women might come from cultures that don’t support contraceptive use. The instability and relocation of the refugee communities could affect the continuous use of contraceptive methods initiated, frequent interruption of healthcare services in refugee camps, and prioritization of basic needs, such as access to food and safety, could also hinder family planning programs [27]. Furthermore, the low educational attainment reported among refugee women in this study (34.3%) and their partners’ low level of education (37.0%) might have affected the consistent use of contraceptive methods.

The main reasons reported for contraceptive discontinuation include desire to become pregnant, experiencing side effects, fear of side effects, husband disapproval, and lack of/no access to the services. Various reasons have been cited for contraceptive discontinuation [28–30]. According to a study by Daniels and colleagues, the most frequent reason given for discontinuing contraception was method-related side effects [28]. Similarly, a recent study identified contraceptive side effects and changes in menstrual cycles as the main reasons for discontinuation [30]. Additionally, it has been reported that women who do not take their pills on a regular schedule are more likely to forget doses [31]. Moreover, the lack of provision of counselling services and dissatisfaction with FP services were identified as the primary factors influencing LARC discontinuation in a systematic review and meta-analysis of 20 studies published in SSA [32]. A phenomenological qualitative study in Ethiopia found that inadequate counselling about family planning, partner opposition, and misconceptions contributed to early discontinuation of long-term contraception methods [29].

Furthermore, there was also evidence that women who received multiple counselling sessions continued their hormonal contraceptives at higher rates than women receiving standard care [33].

Consistent with other studies [12,17,25], higher contraceptive discontinuation was documented among SAC acceptors (emergency contraceptive pills, oral contraceptive pills, and injectables compared to LARC acceptors (IUD and implants. A study from Burkina Faso and the Democratic Republic of Congo found that contraceptive methods themselves are a more significant factor in discontinuation than other reasons. Specifically, users of oral contraceptive pills and injectables discontinued at higher rates than users of implants and IUDs [34]. This could be because, with SAC methods such as pills and injectables, users can deliberately decide and stop using the methods without the knowledge of healthcare providers or having to visit a healthcare facility [35,36]. Additionally, the relatively lower discontinuation rate among LARC acceptors may be because they had already decided to use these methods for a longer duration compared to SAC acceptors [37]. Strengthened counselling and continuous follow-up may help prevent early discontinuation by enhancing users’ awareness of side effects and providing prompt method switching choices [38].

The study’s findings indicated that the likelihood of contraceptive discontinuation during the first year of use for a refugee woman residing in an urban area was 83% higher than that of a woman from a rural area. This finding was in contrast to a study conducted in the National Referral Hospital, Kampala, Uganda. Based on the Ugandan study, a higher rate of early discontinuation was associated with the presence of side effects, not getting proper counseling, and being a rural resident [39]. Unlike rural settings, migrants in urban areas may gain an advantage from contraceptive providers’ accessibility to local suppliers. Living in urban areas may therefore increase access to contraceptive options and decrease unmet needs [40]. However, our finding was consistent with the results documented in Egypt [36]. The incidence of contraceptive discontinuation within 12 months was high among women who live in urban areas of Egypt and those in the highest wealth quintile. The possible reason mentioned was that urban residents expect family planning services to be of the highest quality. Therefore, such women might tend to discontinue the methods soon after they experience side effects and perceived poor quality of services. Similarly, contraceptive methods and service-related reasons, in the Egyptian study, (17%) contributed to the high contraceptive discontinuation among urban residents [36].

Another important determinant of contraceptive discontinuation identified in this study was the length of stay in refugee camps. The study revealed that a one-year increase in the length of stay in the refugee camp for a refugee woman results in a 5% lower risk of contraceptive discontinuation. This finding was not consistent with a study in North Kivu, DRC, where the duration of displaced years had no statistical significance with 12-month contraceptive continuation [17]. The reasons for lower contraceptive discontinuation/ consistent use of the methods/ among women who stayed longer in refugee camps might be related to their stable refugee life and improved relationship with healthcare providers.

The results of the study also demonstrated that women whose husbands were employed in the private sector had a 51% lower likelihood of early contraceptive discontinuation compared to women whose husbands were not employed. However, according to a study on factors associated with oral contraceptive discontinuation in rural Bangladesh, the husband’s occupation was not statistically associated with contraceptive discontinuation [41]. Compared to those who are not employed, employed partners may have higher educational qualifications and are better informed about family planning. They are therefore more supportive of their wives’ continued use of contraceptive methods, and this can be one major reason why women whose partners were employed had less early contraceptive discontinuation. In addition, partner occupation might impact contraceptive use due to time constraints, or economic uncertainty, work-related mobility that affects shared decision-making [42].

Furthermore, our study revealed that refugee women who had an intention to use contraception in the future had an 85% lower risk of contraceptive discontinuation within the first year of use, as expected. Women who have the intention to use contraceptives in the future are typically those who have awareness about family planning, have reproductive goals, and are motivated to prevent unintended pregnancies. Similarly, a study using DHS data in Ethiopia [43] revealed that women who had no clue about their fertility intention had a higher likelihood of discontinuing these methods. In addition, a study by EJ Modey [44] documented that Ghanaian women who have a desire to limit their family size are more motivated to reach this intended fertility goal and are less likely to discontinue the contraceptive method they have initiated.

Our study has limitations worth noting. First, one refugee camp from Gambella was excluded due to safety and security concerns when traveling to the study participants’ residential areas. This could have affected the representativeness of the sample of refugee camps identified for the study. However, in terms of country of origin and culture, the populations in the excluded refugee camp were similar to those included in our study. Second, we had to exclude refugee women for whom there were incomplete records. Information that was not included in the study might have affected our findings. Third, because of the retrospective nature of our study, women may not be able to remember the exact dates of discontinuation and method switch, which could lead to recall bias. However, we maintained privacy and confidentiality during interviews and utilized professional interviewers with standardised questionnaires to encourage open discussion. Furthermore, almost all FP clients registered in the health facilities and participated in this study were married women. As a result, the findings cannot be applied to single refugee women.

## Conclusion

In conclusion, our study documented high early contraceptive discontinuation among married women in selected refugee settings in Ethiopia, with a higher discontinuation rate among short-acting compared to long-acting contraceptive acceptors. Desire to become pregnant, experience and fear of side effects, husband disapproval, and lack of access to the services were the main reasons reported for early contraceptive discontinuation. Short-acting contraceptive acceptors, urban dwellers, short length of stay in the refugee camp, women whose husbands were not employed, and women who did not intend to use contraceptives in the future had a higher likelihood of contraceptive discontinuation within 12 months of use.

High contraceptive discontinuation in refugee women, coupled with low contraceptive utilization, is a critical challenge in addressing the reproductive health needs of women in hard-to-reach situations. To decrease the unmet needs and unintended pregnancies and subsequently lower maternal and child mortality in humanitarian settings in Ethiopia, we recommend that program planners, UNHCR, the higher officials at the MOH of Ethiopia, and RRS give due attention to modern contraceptive services provision and consistent use of the methods in humanitarian settings.

In addition, Family planning service provision in the refugee community should place more emphasis on SAC acceptors, urban residents, women whose partners are unemployed, women who stayed a short time in the camps, and those women who didn’t intend to use contraceptive methods in the future. We recommend both women and their partners take part in contraception counseling, and emphasis should be given to the correct use of the contraceptive methods and side effects of contraceptives during counseling sessions. Finally, we recommend further mixed-methods research to investigate the socio-cultural determinants of method-specific contraceptive discontinuation among refugee women after the first year and subsequent years of use.

## Supporting information

S1 File English version of the questionnaire. (PDF)

S1 Checklist Inclusivity in global research. (DOCX)

S1 MinimalDataset Minimal dataset underlying the findings of the study. (XLSX)
