# Supplementary material for: Early contraceptive discontinuation and associated factors among married women initiating long-acting and short-acting contraceptives in humanitarian settings in Ethiopia: A retrospective cohort study
Source: PLoS One. 2026 Mar 27;21(3):e0345855. doi: 10.1371/journal.pone.0345855 (PMC13028423; doi:10.1371/journal.pone.0345855)
Supplement: S1 File — (PDF) [file pone.0345855.s001.pdf]

S1. English Version of the Questionnaire

1. Name of refugee camp________________ 2. Questionnaire code ____________

3. Name of health facility________________ 4. Interviewer code______________

Part One; Respondents' Socio-demographic information

S.No	Questions	Alternative /choice of response	code	Skip
101	How old were you at your last birthday?	Age in completed years _____		
102	What was your age at the time of initiation of contraception?	________		
103	Have you ever attended school?	Yes ………………………...1<br>No …………………………0		If No<br>Skip to 105?
104	What is your educational level?	No formal education…..….1<br>Primary…………………….2<br>Secondary………………….3<br>Higher……………………..4		
105	To which religion do you belong?	Muslim……………….1<br>Orthodox…………..…2<br>Protestant…………….3<br>Other…………………4<br>Specify__________		
106	Country of origin	Somalia………………….1<br>Eritrea…………………...2<br>South Sudan…………... 3<br>Other…………………….4<br>specify___________		
107	Displaced year	Dd/mm/yyyy		
108	For how long did you stay in the refugee camp?	Year_______<br>Months______		
109	Region	Somali region…………………...1<br>Gambella region………………….2		
110	Residence	Urban………………………...1<br>Rural…………………………0		
111	What is your occupation?	Not working……………1<br>Housewife ………………2<br>Gov't employee…………3		

		Private employee……….4<br>Farmer…………………...5<br>Merchant………………...6<br>Other……………………7<br>Specify___________		
112	How much is your monthly income	__________birr		
113	Wealth Index	Poorest……………..1<br>Poorer ………………2<br>Middle……………….3<br>Richer……………….4<br>Richest………………5		
114	What is your marital status at time of contraception use?	Married and living with husband….1<br>Married not living with husband…...2<br>Not married and living with partner.3<br>Divorced ....4<br>Widowed.......................................5<br>Never married ....6<br>Other ……………………………7<br>Specify_______		
115	What is the highest grade your husband completed?	No formal education…...….1<br>Primary…………………….2<br>Secondary………………….3<br>Higher…………………….4		
116	What is your partner occupation?	Not working……...1<br>Farmer…….………2<br>Merchant …………3<br>Day labor………….4<br>Gov't employee…...5<br>Private employee….6<br>Other………………7<br>Specify_________		
117	Total members of Household	_____________person		

Part Two; Birth/ Reproductive History /Information

S.No	Questions	Alternative /Choice of response	Code	Skip
201	Have you ever been pregnant?	Yes ………………………...1		If no, skip to 209

		No ………………………….0		
202	Number of Pregnancies/gravidities?	________		
203	Have you ever had a live birth?	Yes ………………………...1<br>No ………………………….0		If no, skip to 209
204	Number of live births	-----------------		
205	Did you have a baby in the last one year?	Yes ………………………...1<br>No ………………………….0		
206	How many children do you have?	0…………………………….1<br>1…………………………….2<br>2…………………………….3<br>3…………………………….4<br>4…………………………….5<br>More than 4.........................6		
207	How many are boys?	0…………………………….1<br>1…………………………….2<br>2…………………………….3<br>3…………………………….4<br>4…………………………….5<br>More than 4.........................6		
208	How many are girls?	0…………………………….1<br>1…………………………….2<br>2…………………………….3<br>3…………………………….4<br>4…………………………….5<br>More than 4.........................6		
209	Are you pregnant now?	Yes ………………………...1<br>No ………………………….0		If yes, skip to 211
210	(If no) how long ago did you have your last menstrual period?	Days ____<br>Weeks____<br>Months ___		Skip to 212
211	(IF yes)<br>How many months pregnant are you?	Months ___		
212	At the time you became pregnant did you plan to become pregnant then<br>OR did you want to wait till later<br>OR did you want no more children?	Then…………………………….1<br>Later…………………………….2<br>Not at all/no more children ……3		
213	Have you ever had a pregnancy that miscarried,	Yes ………………………...1<br>No ………………………….0		If no, skip to 215
214	How many of such pregnancies that miscarried have you had?	________________		
215	Have you ever had a pregnancy that aborted?	Yes ………………………...1<br>No ………………………….0		If no, skip to 217
216	How many of such pregnancies have you had?	_____________		

217	Have you ever had a pregnancy that ended in a stillbirth?	Yes ………………………...1<br>No …………………………0		If no, skip to 219
218	How many of such pregnancies that ended in a stillbirth have you had?	____________		
219	Did you visit a health facility during your most recent pregnancy?	Yes ………………………...1<br>No …………………………0		If no, skip to 301
220	When did you seek care at the health facility?	1st trimester…………1<br>2nd Trimester………...2<br>3rd Trimester…………3<br>4th Trimester ……..….. 4		

Part Three; History of Contraceptive Methods Utilization

**S.No**	Questions	Alternative /Choice of response	Code	Skip
301	Have you ever heard of any contraceptive methods?	Yes ………………………...1<br>No …………………………0		If no, END
302	If yes for Q301, which kind of contraceptive method have you ever heard of? (You can answer more than one).	Pills ……………………… 1<br>IUCD……………………… 2<br>Inject able………………… 3<br>Implants ………………….. 4<br>Male condom……………… 5<br>Others ……….…………… 6<br>Specify ______________		
303	From where do you heard the information?	Health facility…………….1<br>Community health worker.2<br>Radio…………………….3<br>TV……………………….4<br>Husband/partner………….5<br>Friends/family member……6<br>Community………………7<br>Other mass media……….8<br>Others…………………..9<br>Specify___________		
304	Where do you get access for contraception	Hospitals………..…1<br>Health center………2<br>Health post……..…...3<br>Private clinics……...4<br>Others………………5<br>Specify___________		
305	Are you using any contraceptive method currently?	Yes ………………………...1<br>No …………………………0		If no, skip to 308

306	If yes, what is/are the methods?<br>More than one option is possible	Female sterilization………………..1<br>Male sterilization…………………..2<br>Pill………………………………….3<br>IUD………………….......………4<br>Injectables…………………………5<br>Implants……………………………6<br>Male Condom……………………...7<br>Female condom…………………...8<br>Diaphragm………………………...9<br>Foam/Jelly……………..................10<br>Lactational Amenorrhea…………11<br>Rhythm/Calendar…………………12<br>Withdrawal………………………13<br>Emergency contraceptive………...14<br>Other……………………………...15<br>Specify________________________		
307	What is the type of contraceptive method used in the last 12 months?<br>More than one option possible	Female sterilization………………1<br>Male sterilization………………….2<br>Pill………………………………...3<br>IUD………………….......………4<br>Injectables…………………………5<br>Implants……………………………6<br>Male Condom……………………...7<br>Female condom…………………...8<br>Diaphragm………………………...9<br>Foam/Jelly……………..................10<br>Lactational Amenorrhea…………11<br>Rhythm/Calendar…………………12<br>Withdrawal………………………13<br>Emergency contraceptive………...14<br>Other……………………………...15<br>Specify________________________		Skip to 311
308	If no to contraception use currently, what is the reason?	Currently pregnant…………1<br>Expecting pregnancy……….2<br>Fear of Side effects…………3<br>Experienced side effects……4<br>Inconvenient to use…………5<br>Infrequent sex/no sex……….6<br>Menopausal/had hysterectomy……………….7<br>Subfecund/infecund………..8<br>Wants as many children as possible……………………9<br>Breastfeeding………………10<br>Other……………………….11<br>Specify______________________		If the respon se is not 3 or 4 skip to 311
309	If the reason for not using contraception currently is side effects? Which side effects	Irregular bleeding……………1<br>No monthly bleeding………...2<br>Heavy/prolonged bleeding……………………….3<br>Unexplained bleeding……….4<br>Headaches………………...……5<br>Abdominal pain………………6<br>Abdominal bloating………...…7<br>Acne……………………………8<br>Weight change……………….....9<br>Tiredness……………………….10		

		Nausea……………………….…….11<br>Abcess……………………….…….12<br>Expulsion…………………….……….13<br>Palpitations/Chest pain……………………………...14<br>Other……………………………15<br>Specify______________________		
310	Which side effect did you experience?	Irregular bleeding…………..…1<br>No monthly bleeding………....2<br>Heavy/prolonged bleeding……………………….3<br>Unexplained bleeding……….....4<br>Headaches……………….....……5<br>Abdominal pain………………..….6<br>Abdominal bloating……….....…7<br>Acne……………………………8<br>Weight change…………..……...9<br>Tiredness……………………….10<br>Nausea……………………….…….11<br>Abcess……………………….…….12<br>Expulsion…………………….……….13<br>Palpitations/Chest pain……………………………...14<br>Other……………………………15<br>Specify______________________		
311	Baseline method used continously12-18 months?	Yes ………………………...1<br>No …………………………0		If yes, 325
312	If no to continuous use, did you switch to another modern method	Yes ………………………...1<br>No …………………………0		If no, 322
313	Switched to another modern method	No………………….…1<br>Yes, in the same month…2<br>Yes, after 1 month………3		If no, 322
314	If yes to method switch, switched to what method?	Female sterilization……………….1<br>Male sterilization………………….2<br>Pill………………………………...3<br>IUD…………………........………4<br>Injectables…………………………5<br>Implants……………………………6<br>Male Condom……………………...7<br>Female condom………………….....8<br>Diaphragm………………………...9<br>Foam/Jelly……………..................10<br>Lactational Amenorrhea…………11<br>Rhythm/Calendar…………………12<br>Withdrawal……………………….13<br>Emergency contraceptive………...14<br>Other……………………………...15<br>Specify________________________		
315	The reason for method switch?	Fear of side effect………………1<br>Inconvenient to use ……………...2<br>Experienced side effects…………3<br>Wanted to try new method………4<br>Advised to switch…………………5<br>Other………………………………6		If the respon se is not 5,

		Specify___________		skip to 317
316	If the reason for method switch was advice, who advised you?	Health worker…………………1<br>Husband……………………….2<br>Friends………………………...3<br>Other family member………..4<br>Other …………………………5<br>Specify___________________________<br>_		
317	Date of initiation of the current/second method	DD/MM/YYYY		
318	Are you still using the second method?	Yes ………………………...1<br>No …………………………0		If yes, 325
319	If no for Q318, for how long did you utilize the contraceptive method?	……………………(in month)		
320	If no for Q318, why are you not using the second method?	Currently pregnant………..1<br>Expecting Pregnancy……….2<br>Fear of side effects………….3<br>Experienced side effects…….4<br>Inconvenient to use………….5<br>Wanted to try a new method…6<br>Advised to stop……………….7<br>Other………………………….8<br>Specify__________		If the respon se is not 7, skip to 323
321	If you were advised to stop, by whom?	Health worker…………………….1<br>Husband……………………..….2<br>Friends…………………………….3<br>Other family member……….….4<br>Other………………………………5<br>specify ____________________		skip to 323
322	If no for Q312, for how long did you use the baseline method?	……………………(in month)		
323	If no to continuous use, did you stop using a modern method all together (abandonment)?	Yes ………………………...1<br>No …………………………0		If no, skip to 325
324	If yes to abandonment, what was the reason?	Desired pregnancy……………1<br>Side effect…………………….2<br>Partner disliked……………....3<br>Family disliked……………….4<br>Became pregnant…………….5<br>Rumours/method bad for her…6<br>Others ……………………….7<br>specify ____________________		

325	Have you ever become pregnant while using contraceptive method?	Yes ………………………...1<br>No …………………………0		If no, skip to 327
326	If yes, while using which method of contraception?	Female sterilization……………….1<br>Male sterilization………………….2<br>Pill……………………………...3<br>IUD………………….......………4<br>Injectables…………………………5<br>Implants……………………………6<br>Male Condom……………………...7<br>Female condom…………….….....8<br>Diaphragm………………………...9<br>Foam/Jelly……………................10<br>Lactational Amenorrhea………….11<br>Rhythm/Calendar…………………12<br>Withdrawal……………………….13<br>Emergency contraceptive………...14<br>Other……………………………...15<br>Specify________________________		
327	Desire for more children	1. Within 2 years<br>2. After 2 years<br>3. Wants no more children		
328	Where did you initiate your current method of contraception	Health center……………1<br>Hospital………………….2<br>Home…………………….3<br>Other…………………….4<br>specify_________________		
329	Your contraceptive method use accepted by your partner?	Yes……………1<br>No……………..2		
330	Partner aware contraception use	Yes……………1<br>No……………..2		
331	Partner approves contraception use	Yes……………1<br>No……………..2		
332	Decision to start contraception	Mainly women…….1<br>Mainly husband…….2<br>Jointly……………….3<br>Health Professional.….4<br>Others…………………5<br>specify_________________		

Part four: Role of Partner and Counseling Services

401	Did you get counseling services before initiating the method?	Yes ………………………...1<br>No …………………………0<br>I don't remember…………. 9		If 0 & 9, skip to 408

402	If yes, what type of counseling did you obtain?	Individual counseling…….1<br>Mass counseling …. …2<br>With husband together….. 3<br>Other ……….…………4<br>Specify_____________		
403	Were you told about how to use the contraceptive method?	Yes ………1<br>No……….. 0<br>I don't remember…9		
404	Were you told about any side effects of the contraceptive method?	Yes ………1<br>No…………0<br>I don't remember…9		If 0 & 9, skip to 407
405	Were you informed about what to do when you experienced these side effects?	Yes ………1<br>No…………0<br>I don't remember…9		
406	Were you told about other forms of family planning you can use if you experienced side effects?	Yes ………1<br>No…………0<br>I don't remember…9		
407	Did the health provider respond to all of your questions?	Yes ………1<br>No……….. 0<br>I don't remember…9		
408	Did you know of any side effects of the method before you begun using it?	Yes ………1<br>No…………0<br>I don't remember…9		If 0 & 9, skip to 413
409	Which side effects did you know of? (More than one option)	Irregular bleeding…………..…1<br>No monthly bleeding………...2<br>Heavy/prolonged bleeding………………………3<br>Unexplained bleeding………...4<br>Headaches………………...….5<br>Abdominal pain………………6<br>Abdominal bloating……….....7<br>Acne…………………………8<br>Weight change………..………9<br>Tiredness……………………10<br>Nausea……………………..…11<br>Abcess…………………….….12<br>Expulsion…………………..…13<br>Palpitations/Chest pain…………………………14<br>Other…………………………15<br>Specify______________________		
4010	How do you know about them?	TV……………………………1<br>Radio…………………………2<br>Health professional…………..3<br>Friends……………………….4<br>Husband…………………..…5<br>Family member…………..…6<br>Others……………………7<br>Specify______________		

411	Did you experience any side effects?	Yes ………1<br>No…………0		If no, skip to 413
412	Which side effects did you experience (More than one option)	Irregular bleeding……………1<br>No monthly bleeding………...2<br>Heavy/prolonged bleeding………………………3<br>Unexplained bleeding………...4<br>Headaches………………...….5<br>Abdominal pain………………6<br>Abdominal bloating……….....7<br>Acne…………………………..8<br>Weight change…………………9<br>Tiredness……………………10<br>Nausea……………………..…11<br>Abcess…………………….….12<br>Expulsion…………………..…13<br>Palpitations/Chest pain…………………………14<br>Other…………………………15<br>Specify______________________		
413	After the initiation of the method, did they appoint you at a specific time?	Yes ………………………...1<br>No …………………………0<br>I don't remember…………9		
414	Did you get follow up counseling service after initiating the method?	Yes ………1<br>No…………0		
415	Did you first discuss with your partner exactly about the method?	Yes ………………………...1<br>No …………………………0<br>I don't remember………….9		
416	Did the health provider respond to all of your questions?	Yes ………1<br>No……….. 0<br>I don't remember…9		
417	Quality of information/counseling received from the provider?	Good……………1<br>Neutral…………2<br>Poor …………….3		
418	Were you been satisfied by the facility cleanliness?	Yes ………1<br>No……….. 0		
419	Were you been satisfied by the health providers' friendliness?	Yes ………1<br>No……….. 0		
420	Were you been satisfied by the health facilities waiting time?	Yes ………1<br>No……….. 0		
421	Did the healthcare provider tell you that everything you discuss would remain confidential?	Yes ………1<br>No……….. 0		
422	Did the healthcare provider respect your opinion and decisions even if they were different from his/hers?	Yes ………1<br>No……….. 0		

423	Do you believe that others could hear your discussions with the health care provider when you were in the FP counseling/ consultation room?	Yes ………1<br>No……….. 0		
424	Did you feel that the healthcare provider showed you respect?	Yes ………1<br>No……….. 0		
425	Complete satisfaction with services received?	Very satisfied……….1<br>Satisfied………………2<br>Neutral……………….3<br>Dissatisfied……………4<br>Very dissatisfied……….5		
426	Distance from Health center/facility? (Km)	……………………km		
427	Do you intend to use contraceptive methods in future?	Yes ………1<br>No…………0		If no, skip to 430
428	Which method would you consider using? (More than one option)	Female sterilization……………….1<br>Male sterilization………………….2<br>Pill………………………………...3<br>IUD……………………......………4<br>Injectables…………………………5<br>Implants……………………………6<br>Male Condom……………………...7<br>Female condom……………….…...8<br>Diaphragm………………………...9<br>Foam/Jelly……………..................10<br>Lactational Amenorrhea…………11<br>Rhythm/Calendar…………………12<br>Withdrawal……………………….13<br>Emergency contraceptive………...14<br>Other……………………………...15<br>Specify_______________________		
429	Why would you intend to use contraceptive methods in future? (More than one option)	Want to limit child bearing……1<br>What to space child bearing…...2<br>Other………………………….3<br>specify___________________		
430	Why would you intend to not use contraceptive methods in future? (More than one option)	Want to have more children…………1<br>Fear of how methods are used……….2<br>Fear of experiencing side effects…….3<br>Husband disapproval…………………4<br>Other………………………………….5<br>Specify_____________________________		

Quality of information(a)_Includes responding yes that the provider explained how to use the contraceptive method, told her about possible side effects, explained what to do in case of side effects, and responded to all of her questions.

Satisfaction (b)-includes being satisfied or very satisfied with the facility's cleanliness, the providers' friendliness, the amount of time spent at the facility, the privacy during her time with the provider, the care she received, the respect shown to her by the provider and the expectation that the provider will keep her information secret.

Part five: Outcome measuring variables (Reasons for discontinuation)

S.N	Questions	Alternative /Choice of response	Skip
501	Date of contraception started (baseline method; 12-18 months before)<br>**From the FP registration book**	Dd/mm/yyyy	
502	Which baseline contraceptive method?	Female sterilization……………….1<br>Male sterilization………………….2<br>Pill……………………………....3<br>IUD…………………......………4<br>Injectables…………………………5<br>Implants……………………………6<br>Male Condom……………………...7<br>Female condom…………………..…...8<br>Diaphragm………………………...9<br>Foam/Jelly……………..................10<br>Lactational Amenorrhea…………11<br>Rhythm/Calendar…………………12<br>Withdrawal……………………….13<br>Emergency contraceptive………...14<br>Other……………………………...15<br>Specify________________________	
503	Are you still using the baseline contraceptive method?	Yes ………………………...1<br>No …………………………0	If yes to 503, End section 5
504	If no for Q503, for how long did you utilize the contraceptive method?	……………………(in month)	
505	Specific date of discontinuation/removal (probe)	Dd/mm/yyyy	
506	What was the reason for the discontinuation of the baseline method?<br>(More than one option)	Infrequent Sex/ Husband away……. 1<br>Became pregnant while using….…. 2<br>Wanted to become pregnant ……….3<br>Husband disapproved ………………4<br>Wanted more effective method …….5<br>Health concerns …………………….6<br>Fear of Side effects …………………7<br>Side effects experienced ……………8<br>Lack of access/too far ………………9<br>Cost too much ……………………..…10<br>Inconvenient to use ……………….…11<br>Difficult to get pregnant/ menopause. 12<br>Marital dissolution/separation ………13<br>Don't know …………………………14<br>Others……………………..………15<br>Specify_______________________	
507	Place of discontinuation/removal?	Health center…...1<br>Hospital…………2<br>Health post………3<br>Others……………4<br>Specify_________	

Thank you!
