# Supplementary material for: Early contraceptive discontinuation and associated factors among married women initiating long-acting and short-acting contraceptives in humanitarian settings in Ethiopia: A retrospective cohort study
Source: PLoS One. 2026 Mar 27;21(3):e0345855. doi: 10.1371/journal.pone.0345855 (PMC13028423; doi:10.1371/journal.pone.0345855)
Supplement: Attachment — Submitted filename: Peer review comments.docx [file pone.0345855.s004.docx]

# **Detailed Peer‑Review Report for “Early contraceptive discontinuation and associated factors among married women initiating long‑acting and short‑acting contraceptives in humanitarian settings in Ethiopia”**

## **Overview and significance**

The study under review aims to quantify and explore the determinants of **first‑year contraceptive discontinuation** among married refugee women in Ethiopia. Using a retrospective cohort design, the authors followed 551 women who initiated either long‑acting reversible contraceptives (LARC) or short‑acting methods (SAC) and measured discontinuation, switching and abandonment. Discontinuation rates were high (overall 41.2 %; SAC 44.9 % vs LARC 32.3 %), and multivariable Cox models identified method type, residence, husband’s occupation, length of stay in camp and future contraceptive intention as significant predictors of discontinuation. This topic is important because reproductive‑health services for displaced populations are often neglected; understanding method dynamics can inform family‑planning programmes in humanitarian settings.

## **Major comments by section**

### **Title, and abstract (lines 1–43 and 25–83)**

The title clearly states the population, exposure and outcome of interest. The abstract provides a concise summary of the background, methods, results and conclusions. However, the conclusions repeat statistics already given in the results (lines 60–69). Consider using the abstract’s conclusion to highlight implications and recommendations rather than repeating the discontinuation rate. Additionally, define LARC and SAC at first mention and avoid acronyms without definition (lines 42–43).

### **Introduction (lines 85–146)**

- **Contextualisation and citation** – The introduction summarise the scale of the refugee crisis in Ethiopia and the importance of contraception for displaced women (lines 87–104). It references global discontinuation rates and highlights that discontinuation leads to adverse outcomes (lines 105–110), which provides context. Nonetheless, several statements lack citations. For example, the claim that contraception reduces maternal morbidity and mortality (lines 96–103) should be supported by evidence. Similarly, statistics on discontinuation rates and method differences (lines 112–119) should be attributed to specific studies or meta‑analyses. Providing current references (2022–2025) would strengthen the introduction.
- **Rationale and research gap** – The authors assert that evidence on contraceptive use dynamics in humanitarian settings is scarce (lines 133–143). This gap is well framed, but it would be useful to mention recent reports or WHO guidance on family‑planning in crisis contexts to underscore the study’s novelty. Clarify why only married women were included and how findings might differ for unmarried women.

### **Methods**

#### **Study design, setting and participants (lines 148–176)**

- **Design classification** – The study is described as a “retrospective follow‑up study” (lines 148–153). As data on initiation and discontinuation were collected at one time point using retrospective recall, the design is essentially a **retrospective cohort**. Clarify this in both abstract and methods and acknowledge the potential for recall bias when women report discontinuation dates.
- **Study setting description** – The manuscript provides a detailed description of the refugee camps and explains why some camps were excluded for security reasons (lines 162–171). However, to assess generalisability, please provide basic demographic characteristics of the excluded camp or explain how refugees there may differ from those included. Explain why only Somali and South Sudanese camps were selected and whether cultural differences could influence discontinuation.

#### **Sample size and sampling (lines 182–223)**

- **Sample‑size calculation** – The sample size is calculated using a formula for survival analysis based on two proportions and a hazard ratio (lines 184–200). The formula and variables are not clearly formatted in the manuscript – the denominator of the formula and the source of the hazard ratio (HR = 1.74) are not explained. Please rewrite the formula in a readable format (e.g., as an equation) and justify the choice of HR = 1.74 and discontinuation probability of 18.4 % by citing the previous Congo study. As currently written, the derivation of 566 participants is difficult to follow.
- **Sampling procedure** – All eligible women with complete records were included (lines 207–225), resulting in 551 participants. Provide counts for the women excluded due to incomplete records and specify whether excluded women differed from those included. Clarify how many camps were initially sampled and how the lottery method was implemented.

#### **Data collection and quality assurance (lines 232–247, 281–291)**

- **Instrument development** – The questionnaire was adapted from existing literature and translated into local languages (lines 240–244). Include a brief summary of the main items (e.g., socio‑demographics, contraceptive history, partner variables) and refer to the supplementary file. Describe any pilot testing of the tool.
- **Data quality** – The authors indicate that pre‑testing and extensive training were conducted and that data was collected electronically (lines 283–292). This is commendable. However, recall bias may still be present because discontinuation and switching dates were self‑reported. Acknowledge this limitation explicitly and consider cross‑checking with clinic records if possible.

#### **Variable definitions (lines 253–279)**

- **Outcome definitions** – The definitions of “continuer,” “switcher” and “discontinuer” are clearly stated (lines 263–270). Nevertheless, this categorisation may misclassify women who temporarily discontinued and later restarted a method. Clarify whether women who switched to a different modern method were considered discontinuers or censored at the time of switch. Provide a rationale for limiting the discontinuation observation window to 12 months.
- **Exposures and covariates** – The manuscript lists socio‑demographic and reproductive variables but does not specify how variables like wealth index were derived (lines 256–259). Briefly describe the construction of the wealth index (e.g., principal component analysis on assets) and coding of categorical variables. For partner awareness and approval, specify the questions asked and the response options.

#### **Statistical analysis (lines 294–320)**

- **Candidate selection** – The authors used log‑rank tests to identify covariates with p < 0.25 for inclusion in the Cox model (lines 303–314). Relying solely on univariate p‑values may omit important confounders. Consider using conceptual frameworks or prior literature to select covariates. Variables such as education, parity and fertility desire are known predictors of discontinuation but were excluded because they lacked univariate significance.
- **Proportional hazards assumption** – The methods state that proportional hazards (PH) assumptions were checked (lines 316–318) but no details are provided. Survival analysis literature emphasises that validating the PH assumption is crucial; graphical diagnostics and goodness‑of‑fit tests should be reported. Provide details on the tests used (e.g., Schoenfeld residuals, log‑minus‑log plots) and describe whether any variables violated the assumption. If violations were found, consider using stratified or time‑varying covariates.
- **Handling of clustering and censoring** – Refugee women within camps may share unobserved characteristics. Describe whether clustering was assessed and whether robust standard errors were used. Clarify how censored observations (e.g., women still using contraception at 12 months) were handled.

### **Results**

#### **Presentation of descriptive results (lines 347–720)**

- **Tables and formatting** – Tables 1–4 are presented in plain text with extensive repetition of parentheses and percentages (lines 360–720). The formatting makes them difficult to read. Reformat tables to clearly list categories, counts and percentages with footnotes explaining abbreviations. Combine categories where appropriate (e.g., group age categories). Consider summarising key findings in the text and moving detailed tables to supplementary material.
- **Interpretation of descriptive statistics** – The results section reports many comparisons between LARC and SAC users with p‑values but does not interpret their practical significance. For example, LARC users were more likely to have a longer mean stay in camp and to be urban residents (lines 360–420). Discuss why these differences might influence discontinuation rather than simply presenting p‑values.

#### **Contraception dynamics (lines 720–840)**

- **Discontinuation rates** – The authors report discontinuation rates at three, six and twelve months and list reasons for discontinuation (lines 720–732). Present confidence intervals for these estimates. When comparing reasons for discontinuation between methods, use proportions rather than raw numbers to aid interpretation. Clarify how many women experienced method failure and whether pregnancy outcomes were recorded.
- **Switching and abandonment** – Table 4 summarises switching and abandonment (lines 724–740). Clarify whether switching was considered a competing event or treated as censoring in the survival analysis. The term “abandonment” should be clearly defined earlier (lines 272–274) and its distinction from discontinuation explained.

#### **Determinants of discontinuation (lines 840–960)**

- **Model output** – The Cox regression table (Table 5) provides crude and adjusted hazard ratios. Ensure that the reference categories are clearly stated. Note that some variables (e.g., wealth index, number of children) show attenuated associations in the adjusted model, suggesting confounding. The finding that urban residence and short camp stay are associated with higher discontinuation is interesting and warrants deeper exploration. Report model diagnostics (e.g., Akaike information criterion) and goodness‑of‑fit statistics.
- **Interpretation of hazard ratios** – Use correct terminology when interpreting hazard ratios; they estimate instantaneous risk rather than absolute risk. For continuous variables like length of stay, specify the unit of increase (1 year). Acknowledge that the hazard ratio of 1.50 for SAC users indicates a 50 % higher instantaneous hazard compared with LARC users, but cumulative risk differences may differ over time.

### **Discussion and conclusion (lines 960–1089)**

- **Comparison with literature** – The discussion compares the discontinuation rate to studies from Uganda, Congo and Myanmar (lines 960–975). Broaden the comparison to include recent DHS meta‑analyses or systematic reviews on discontinuation. When comparing rates, consider differences in population (married vs all women), recall periods and method mix. Avoid speculative explanations without evidence; for example, attributing higher discontinuation among refugees to “unstable life and relocation” (lines 990–1004) should be supported by data or qualitative insights.
- **Mechanisms and implications** – The manuscript hypothesises that SAC users discontinue because they can stop without visiting a clinic (lines 997–1002). Provide references to support this and discuss how counselling and follow‑up could mitigate discontinuation. The observation that urban residents have higher discontinuation is interesting; propose mechanisms (e.g., quality expectations, service interruptions) and consider alternative explanations. Discuss how partner occupation influences discontinuation and whether male engagement programmes might help.
- **Limitations** – The authors acknowledge exclusion of one camp and potential recall bias (lines 1074–1091). Additionally, discuss selection bias due to excluding women with incomplete records and the limited generalisability to unmarried women. Recognise the potential for misclassification of discontinuation episodes and for unmeasured confounding. Describe any steps taken to minimise these biases.
- **Conclusions and recommendations** – The conclusion reiterate the high discontinuation rate and recommends prioritising SAC users, urban residents and women with short camp stays (lines 1080–1088). Emphasise that interventions should include improved counselling, easy access to method switching and male partner engagement. Encourage further mixed‑methods research to understand the sociocultural drivers of discontinuation.

## **Minor comments and editorial issues**

1. **Grammar and style** – Several sentences contain typographical errors or awkward phrasing (e.g., “contraception initiations” instead of “contraceptive initiations” and “contraception discontinuation” instead of “contraceptive discontinuation”). Review the manuscript thoroughly for grammar, punctuation and tense consistency. Use present tense for general statements and past tense for study findings.
2. **Formatting of equations** – The sample‑size formula and hazard ratio definitions should be presented using mathematical notation rather than embedded within text (lines 197–201). Use standard notation for logarithms (e.g., $\log$ ) and ensure parentheses are balanced.
3. **Acronyms and abbreviations** – Define all acronyms (LARC, SAC, FP, ANC, ODK) at first use and use them consistently thereafter. Avoid switching between “contraception” and “contraceptives” within the same sentence.
4. **Tables and figures** – Provide captions that explain the content and include units and footnotes explaining abbreviations. Ensure that figures are legible and include number at risk in Kaplan–Meier plots. Consider presenting the reasons for discontinuation as a stacked bar chart to facilitate comparison by method.
5. **References** – Update the reference list to include more recent literature (2022–2025) on contraceptive discontinuation, especially from humanitarian contexts. Ensure that all in‑text citations are properly numbered and correspond to the reference list. Check the format of the reference numbering (e.g., use square brackets consistently) and remove extraneous spaces or punctuation.

## **Strengths of the study**

- **Novel setting** – Conducting a discontinuation study in refugee camps fills a critical gap in reproductive‑health research and highlights challenges faced by displaced women.
- **Large sample and comprehensive data collection** – Including all eligible women from registries and conducting computer‑assisted interviews minimises selection bias and reduces data‑entry errors. The study collected detailed socio‑demographic, reproductive and partner information.
- **Policy relevance** – Identifying modifiable factors such as method type, residence and partner occupation provides actionable insights for humanitarian agencies. The recommendation to enhance counselling and consider partner involvement aligns with WHO guidance on family‑planning programmes.

## **Summary and recommendations**

This manuscript addresses an important issue and provides useful data on contraceptive discontinuation among refugee women. The high discontinuation rates underscore the need for targeted interventions in humanitarian settings. However, the manuscript requires substantial revision to improve clarity, methodological transparency and statistical rigour. Clarify the retrospective cohort design, provide a clear sample‑size derivation, report diagnostic checks for the proportional hazards assumption, and enhance discussion of findings relative to the literature. Improving tables and figures and correcting grammatical errors will also enhance readability. Addressing these issues will strengthen the manuscript’s contribution to the field and increase its suitability for publication.
