# Supplementary material for: Early contraceptive discontinuation and associated factors among married women initiating long-acting and short-acting contraceptives in humanitarian settings in Ethiopia: A retrospective cohort study
Source: PLoS One. 2026 Mar 27;21(3):e0345855. doi: 10.1371/journal.pone.0345855 (PMC13028423; doi:10.1371/journal.pone.0345855)
Supplement: Attachment — Submitted filename: Response to Reviewers.docx [file pone.0345855.s006.docx]

# Rebuttal Letter

Academic Editor

*PLOS ONE*

November 18, 2025

Dear academic editor,

Thank you for inviting us to submit a revised draft of our manuscript entitled "Early contraceptive discontinuation and associated factors among married women initiating long-acting and short-acting contraceptives in humanitarian settings in Ethiopia: A retrospective follow-up study" to *PLOS ONE.* We also value the time and energy you and the other reviewers invested in providing thoughtful feedback on how to strengthen our paper. We are therefore delighted to resubmit our article for further consideration. We have made the required modifications in light of your extensive comments and suggestions. We hope our edits and responses below satisfactorily address all the issues and concerns you and the reviewers have noted.

To facilitate your review of our revisions, the following is a point-by-point response to the questions and comments in a letter dated November 18, 2025

**ACADEMIC EDITOR’S COMMENTS**

First, thank you so much for your precious time, insightful comments, and suggestions for improving our paper.

1. **Academic editor’s comment:** The manuscript needs substantial revision in terms of its placement in the literature, details on the setting and methods, and testing the assumptions of the statistical analysis.

- **Response:** Thank you for your important feedback. We have substantially revised the manuscript to better align with the literature (page 4, lines 93-97), (Page 5-6), Page (22, 1966-1971), clarified the setting and methods (Page 7, lines 167-171), and included the required diagnostic checks for statistical assumptions (Page 12, 339-345).

2. **Academic editor’s comment:** The term “Wealth Index” is misleading; in fertility surveys, it refers to an index based on household assets rather than self-declared perception. Please consider using “Self-declared wealth” or similar

- **Response:** Thank you for your insightful suggestion. We agree with the concern and have changed "Wealth Index" to "Self-declared wealth" throughout the manuscript to avoid confusion.

3. **Academic editor’s comment:** The funding information provided in the ‘Funding Information’ and ‘Financial Disclosure’ sections do not match.

- **Response:** We have corrected and harmonized the grant information in both sections. The grant number for **WHO HRP–University of Ghana is** 2022/1230378.

4. **Academic editor’s comment:** Please provide more information on how verbal consent was documented and whether this was approved by the IRB.

- **Response:** We have revised the Ethics statement (Methods, Ethical consideration part (Page 13, lines 374-379) to explain how verbal consent was documented. After reading the participant's consent statement, the interviewer signed a record confirming that consent was obtained. This procedure was approved by the SPHMMC IRB (Ref no: PM23/401/23/01/2024)

5. **Academic editor’s comment:** Please ensure that your data are available according to PLOS ONE’s data policy.

- **Response:** We have updated the Data Availability statement. The data underlying our findings are now provided within the manuscript and its Supporting Information files, in accordance with PLOS ONE’s data policy.

6. **Academic editor’s comment:** Please ensure that the ethics statement appears only in the Methods section.

- **Response:** Thank you for the clarification. We confirm that the Ethics Statement is already included exclusively in the Methods section of the manuscript, and it does not appear in any other section. No changes were required, but we have revised it to ensure full compliance with PLOS ONE’s requirements.

7. **Academic editor’s comment:** Please include the PLOS inclusivity questionnaire as Supporting Information.

- **Response:** The completed questionnaire has been added as Supporting Information (S2 Checklist)

**REVIEWER 1 COMMENTS:**

We are deeply grateful to Reviewer 1 for the careful reading of our manuscript and for the valuable comments and suggestions, which have greatly improved the overall quality of the paper.

1. **Reviewer 1 comment (Abstract):** Abstract: Is there a reason you switch between RH and SRH in the Background? It seems you could just use SRH consistently.

- **Response:** We thank the reviewer for pointing this out. We have now used the term **“sexual and reproductive health (SRH)”** consistently throughout the abstract and the rest of the manuscript to maintain clarity and uniformity.

2. **Reviewer 1 comment (Introduction):** Lines 65–68: I believe these sentences need references: “Women in humanitarian settings are often unable to find contraceptive methods when only a small number would desire to become pregnant” and “In addition, as a strategy for war and conflicts, women and girls are at increased risk of early and forced marriage, sexual and gender-based abuse, sexual exploitation, and transactional sex.”

- **Response:** We appreciate your observation. We have now added appropriate references to support these statements. The revised version cites [Achola et al., 2024] and [Tanabe et al., 2017] to substantiate the claims regarding limited contraceptive access and the risks faced by women and girls in humanitarian settings.

3. **Reviewer 1 comment (Introduction):** Lines 71–73: Also needs a reference. “Moreover, contraceptive provision can reduce maternal mortality and morbidity from unwanted pregnancies and unsafe abortions and help to prevent sexually transmitted infections from spreading within the population.”

- **Response:** Thank you for this important suggestion. We have now included relevant citations [Ali, M. M., Cleland, J. G., & Shah, I. H., 2012] supporting the evidence that contraceptive provision contributes to reductions in maternal mortality, morbidity, and sexually transmitted infections.

4. **Reviewer 1 comment (Introduction):** Lines 93–97: Please add references for some of these statements.

- **Response:** We agree and have now added several references [Khalifa et al., 2022; Mekonnen & Wubneh, 2020; Sedgh et al., 2014] to strengthen these statements and ensure that all factual claims are properly supported.

5. **Reviewer 1 comment (Introduction):** Line 103: Isn’t reference 13 a study on contraceptive discontinuation in a humanitarian setting?

- **Response:** We thank the reviewer for noting this. We agree that reference 13 found evidence on contraception discontinuation in humanitarian settings. We have revised the sentence and clarified that, apart from a study by Casey in DRC, there remains a critical evidence gap on contraceptive discontinuation, method switching, method failure, and contraceptive abandonment in the humanitarian context.

6. **Reviewer 1 comment (Introduction):** Introduction: Would you please add a brief description of the selected refugee camps? For example, urban vs. rural? Population size? Are contraceptive services available at health facilities in the selected camps?

- **Response:** Thank you to the reviewer for making this thoughtful recommendation. We agree that providing background information on refugee camps improves readers' understanding of the study's context. As a result, we have added a brief description of the camps, including their location (urban/rural classification), population size, and availability of contraceptive services, under the "Study Setting" section (Page 7, lines 167-171), which we believe is a more appropriate place for such contextual and methodological details.

7. **Reviewer 1 comment (Methods)**: Line 131: Is FP provided only to married women? Is there a reason to limit your study population to only married women?

**Response:** We appreciate the reviewer raising this point. In the updated manuscript, we clarify the rationale for focusing on married women. In the study setting, the majority of FP clients at the RRS health center in selected refugee camps were married women, and the health facility registries primarily document information for married women. Furthermore, the sociocultural norms in these refugee camps often associate the use of contraceptives with marriage. As a result, we limited the study population to married women to ensure data accuracy and consistency with current healthcare data. We have included this explanation in the Methods section (Study participants and selection procedure) (Page 8, lines 182-188).

8. **Reviewer 1 comment (Methods)**: Line 197: What do you mean by “resumption of use at post-discontinuation”? Do you mean people who terminated method use without switching?

- **Response:** Thank you for your observations. The term "resumption of use at post-discontinuation" refers to women who stopped using a contraceptive method and then resumed using the same or a different method after a time of nonuse. To ensure clarity, we rephrased the sentence in the updated version (Methods, Study variable subsection) (Page 11, lines 297-298).

9. **Reviewer 1 comment (Methods)**: Methods: Could you say a bit more about how you approached selected women for an interview, particularly since women gave no prior consent to be contacted about their contraceptive use? Presumably, women could feel that the services at the health facility were not confidential if a stranger showed up looking for them to talk about their contraceptive use. You specifically mention in Line 237 that the majority of participants kept their contraceptive use secret. How did you avoid breaking that confidentiality and making sure the fact that you interviewed them did not reveal their contraceptive use to others?

- **Response:** Thank you for your important comment. We have added an explanation in the amended Methods section that states, "Health professionals who were already known to the women first informed eligible participants about the study and sought their verbal consent for the research team to approach them."

As stated in the sampling technique, initial contact was made exclusively through health workers known to the women, interviews were held in the participants' homes, and no identifying information was collected. The IRB examined and approved the approach, as well as the use of verbal informed consent due to the sensitive nature of the issue.

10. **Reviewer 1 comment (Results):** I recommend that you reduce most % and ages to one decimal point (eg, 29.9 rather than 29.93 or 93.3% instead of 93.29%) as you present a lot of data. I also think it would be more clear if you present the %s in the narrative and only show the N’s in the table. I would recommend reducing the wordiness in the Results section. For example, you could also adjust text like in line 271 to say ‘42.1% of LARC and 40.0% of SAC acceptors…’ rather than write out ‘over forty percent’ and ‘nearly forty percent’.

- **Response:** Thank you for your valuable advice. We agree and rounded all percentages and means to one decimal place in the Results and Tables. While we understand the importance of shortening the presentation, we believe it is clearer and more informative to show both numbers and percentages in the tables. Furthermore, the Results section has been substantially changed to improve clarity and minimize wordiness

11. **Reviewer 1 comment (Results):** Line 265: what does 14.74 or 12.04 refer to? the mean number of years?

- **Response:** Thank you for your comment. The numbers (14.74 and 12.04) represent the average length of stay in the refugee camp for LARC and SAC acceptors, as described in the text.

12. **Reviewer 1 comment (Results):** Line 269: all of the refugee camps were not rural? If not, please describe the context of the selected camps in the Introduction.

- **Response:** Thank you for your observation. As per your prior recommendation, the location and context of the refugee camps have already been included in the study setting section under Methods (Page 7, lines 167-171).

13. **Reviewer 1 comment (Results):** Tables: please reduce all % to one decimal place. It would be good to indicate somewhere in the table that the number in parentheses is the % and the other number is the N.

- **Response:** Done. Percentages in tables have been revised to one decimal point. We also indicated values with percentages in the table headings.

14. **Reviewer 1 comment (Results):** Tables: To reduce the size of the tables, any variable with only Yes/No responses, you could report only the Yes.

- **Response:** Thank you for your suggestion. We have updated the tables to include only the "Yes" category for binary variables.

15. **Reviewer 1 comment (Results):** Table 1: I don’t think you need to report mean age at initiation and mean age now since everyone in the sample initiated FP within the previous 12–18 months…

- **Response:** Thank you for this comment. The woman's age at contraceptive initiation refers to the first time she used any contraceptive method in her life, whereas the respondent’s age is her age at the time of the interview. To capture this distinction, we kept both variables.

16. **Reviewer 1 comment (Results):** Table 1: what is the difference between ‘Housewife’ and ‘Not working’ if everyone is married? Should these be collapsed?

- **Response:** Thank you for noticing this. To improve clarity, we combined these two categories into a single variable ("Not working/Housewife").

17. **Reviewer 1 comment (Results):** Line 292 and Table 2: was the desire for more children measured as within 2 years of now or of when they started using the method?

- **Response:** The desire for more children was assessed in relation to the timing of the interview. We clarified this in the paper as a footnote

18. **Reviewer 1 comment (Results):** Line 302: could you say how partner approval was different?

- **Response:** Yes, thank you. We now describe that LARC users had higher partner approval than SAC users.

19. **Reviewer 1 comment (Results):** Line 307 and Table 3: were all participants asked about intention to use contraception in the future or only those who had terminated their contraceptive use?

- **Response:** Thank you. The question on future intention to use contraception was asked of all women to capture both potential use among non-users and continuation intentions among current users, using language that clearly enabled women to indicate whether they intended to continue, switch, or discontinue using contraception.

20. **Reviewer 1 comment (Results):** Line 323: what is the difference between ‘experiencing side effects’ and ‘fear of side effects’? Seems like those could be combined.

- **Response:** Thank you for your comment. We maintained the terms 'fear of side effects' and 'experiencing side effects' separate since fear of side effects is usually linked to misconceptions or peer pressure influencing non-use or discontinuation, whereas experiencing side effects refers to actual method-related complaints that vary among users. Distinguishing these two provides valuable evidence for developing targeted interventions.

21. **Reviewer 1 comment (Results):** Figure 3: Seems like the Y-axis could have a maximum of 100…

- **Response:** Thank you for your comment. The reasons for contraception discontinuation are presented in a stacked bar chart, making it easier to compare methods.

22. **Reviewer 1 comment (Results):** Line 328–329: ‘Among women who discontinued…’ What did the rest who discontinued the method do?

- **Response:** Thank you for your question. A total of 227 women discontinued their baseline method. Of these, 88 stopped using contraception entirely, indicating that they had no plans to use any kind of method again. The remaining women either switched to another method or temporarily discontinued for reasons such as planning to become pregnant or having become pregnant, to resume or restart contraception later, either the same or a different method.

23. **Reviewer 1 comment (Results):** Line 330–331: clarify method failure question.

- **Response:** Thank you for your comment. Because of the small number of women who encountered method failure/got pregnant while using contraceptives/, the method failure question in this study focuses on lifetime experience rather than the reason for current discontinuation. Out of 551 participants, 47 women reported method failure in their lifetime. Of those who experienced method failure (47), 9 women reported while using the baseline method. Method failure is explicitly defined under the study variables in the methods section.

24. **Reviewer 1 comment (Results):** Lines 358–360: intention to use FP for current users.

- **Response:** This has already been explained above.

25. **Reviewer 1 comment (Results):** Table 5: too many categories; small N.

- **Response:** Thank you. Table 5 shows the multivariable Cox regression to identify determinants of time to early contraceptive discontinuation. We kept all categories to maintain meaningful associations, as merging them could hide essential relationships. To validate the model's validity, we verified the proportional hazards assumption with the Schoenfeld residuals test.

26. **Reviewer 1 comment (Results):** Table 5: use “short acting” and “long acting” consistently.

- **Response:** Short-acting and long-acting are now used consistently throughout the manuscript.

27. **Reviewer 1 comment (Results):** Line 380: the Congo study participants were conflict-affected, not refugees.

- **Response:** Thank you for the correction. We changed the text to appropriately describe the Congo study population as conflict-affected rather than refugees.

28. **Reviewer 1 comment (Discussion):** I would recommend that you report fewer % from your data in this section. You’ve already reported them in the Results. For example, lines 404-406, I recommend deleting the numbers by method.

- **Response:** Thank you for your suggestion. We deleted percentages in the "discussion" section to reduce duplication.

29. **Reviewer 1 comment (Discussion):** Since side effects was a common reason for discontinuation, it may be good to reflect on how improvements to contraceptive counseling and information provided could potentially improve continuation. You sort of bring up quality of service delivery in your conclusion but seems like you could address this in the Discussion.

- **Response:** Thank you for your insightful suggestion. We have included a paragraph (paragraph 4; Page 23, lines 2004-2030) in the Discussion section that emphasizes how addressing common reasons for contraceptive discontinuation, notably side effects, as well as providing proper contraceptive counselling and information, could improve method continuation among women.

30. **Reviewer 1 comment (Discussion):** Lines 393-400: You mention multiple reasons for higher discontinuation among refugee women in your sample – do you know if any of these are true? For example, was there a lot of movement among the refugee populations in the selected camps?
31. **Response:** Thank you for your comment. Yes, there was frequent movement among the refugee population. As illustrated in Figure 3, husband away/ infrequent sexual activity was a common cause of discontinuation. Refugee populations frequently return to their country of origin or other places, which may contribute to interruptions in contraceptive use and follow-up.
32. **Reviewer 1 comment (Discussion):** Line 417-420: I am a little confused by the statement here which seems to indicate that continuation should be higher in urban settings? That’s not what you found, though?”

- **Response:** We appreciate the observation. While our findings differ from this pattern, we added the statement to help contextualize our findings in light of previously published information.

33. **Reviewer 1 comment (Discussion):** Line 435: Here, you contradict what you say in Line 397. Here, you say the refugees may be more stable, but in Line 397 you say they’re less stable?

- **Response:** Thank you for highlighting the apparent inconsistency. The two statements refer to distinct concepts. The statement in Line 397 is about population mobility; many refugee women travel frequently (e.g., to their country of origin for family or livelihood reasons), which can disrupt follow-up and contribute to increased discontinuation. Line 435, on the other hand, refers to the length of stay in the camp: women who have been there for a longer period of time are more established and are less likely to discontinue contraception.

34. **Reviewer 1 comment (Discussion):** Lines 443-444:  This statement needs a reference OR you should say they ‘may have’ to soften it since it is your inference, not necessarily a fact.

- **Response:** Thank you for your feedback. Revised accordingly.

35. **Reviewer 1 comment (Discussion):** Line 479-480: Do you have data on contraceptive prevalence among refugee populations in Ethiopia? This would be useful information to present in the Introduction. In general, I think there have been a number of studies on refugees in Ethiopia – could you reference some SRH data on refugees in Ethiopia in the Introduction or Discussion?

- **Response:** Thank you for the suggestion. We have included available **SRH and unmet need prevalence data for refugee populations in Ethiopia** in the Introduction and cited relevant studies.

36. **Reviewer 1 comment (Discussion):** Lines 486-488: I’m not sure what you mean by ‘providers should place more emphasis on’ those specific categories?

- **Response:** Thank you for the comment. We have revised the statement for clarity. It now reads: “Family planning service provision in the refugee community should place more emphasis on…” instead of referring specifically to healthcare providers.

**REVIEWER 2 COMMENTS**

## 1. **Reviewer 2 comment (Overview and significance)**

The study under review aims to quantify and explore the determinants of **first‑year contraceptive discontinuation** among married refugee women in Ethiopia. Using a retrospective cohort design, the authors followed 551 women who initiated either long‑acting reversible contraceptives (LARC) or short‑acting methods (SAC) and measured discontinuation, switching and abandonment. Discontinuation rates were high (overall 41.2 %; SAC 44.9 % vs LARC 32.3 %), and multivariable Cox models identified method type, residence, husband’s occupation, length of stay in camp and future contraceptive intention as significant predictors of discontinuation. This topic is important because reproductive‑health services for displaced populations are often neglected; understanding method dynamics can inform family‑planning programmes in humanitarian settings.

- **Response:** We truly appreciate the reviewer for providing a clear summary and acknowledging the significance of this study. We appreciate the recognition of our work addressing contraceptive discontinuation among refugee women, a population that is frequently underrepresented in reproductive health studies. The comments were well taken, and they helped us improve the manuscript's clarity, organisation, and interpretation.

## 2. **Reviewer 2 Major comments by section:**

### **Title, and abstract (lines 1–43 and 25–83)**

The title clearly states the population, exposure and outcome of interest. The abstract provides a concise summary of the background, methods, results and conclusions. However, the conclusions repeat statistics already given in the results (lines 60–69). Consider using the abstract’s conclusion to highlight implications and recommendations rather than repeating the discontinuation rate. Additionally, define LARC and SAC at first mention and avoid acronyms without definition (lines 42–43).

- **Response:** We appreciate the reviewer’s recommendation. We have updated the conclusion under the abstract section to focus on implications and recommendations. LARC and SAC were already defined at first mention (lines 33-34).

### 3. **Reviewer 2 comment (Introduction) (lines 85–146):**

- **Contextualisation and citation** – The introduction summarise the scale of the refugee crisis in Ethiopia and the importance of contraception for displaced women (lines 87–104). It references global discontinuation rates and highlights that discontinuation leads to adverse outcomes (lines 105–110), which provides context. Nonetheless, several statements lack citations. For example, the claim that contraception reduces maternal morbidity and mortality (lines 96–103) should be supported by evidence. Similarly, statistics on discontinuation rates and method differences (lines 112–119) should be attributed to specific studies or meta‑analyses. Providing current references (2022–2025) would strengthen the introduction.
- **Response**: We appreciate the reviewer's suggestion. In response, we have incorporated recent evidence (2022 and 2024) that we believe is significant and pertinent to our paper. Moreover, we have included relevant reference citations to strengthen the introduction and provide strong support for the points stated.
- **Rationale and research gap** – The authors assert that evidence on contraceptive use dynamics in humanitarian settings is scarce (lines 133–143). This gap is well framed, but it would be useful to mention recent reports or WHO guidance on family‑planning in crisis contexts to underscore the study’s novelty. Clarify why only married women were included and how findings might differ for unmarried women.
- **Response**: We appreciate the reviewer's insightful recommendation. We updated the rationale of the study to reflect how our study aligns with “the World Health Organization's Global Research Agenda on Health, Migration, and Displacement (2023).” This update demonstrates how our research supports the WHO's recommendation to produce context-specific evidence to improve SRH care and guide policy in humanitarian settings (Page 5, lines 139-144). Concerning the inclusion of only married women, we stated in the Methods section that only married women were included since they account for the majority of contraceptive users in the study area and are the primary target group for family planning services in refugee camps. Furthermore, we stated in the Limitations section that the findings may not apply to unmarried women

### 4. **Reviewer 2 comment (Methods)**

#### **Study design, setting and participants (lines 148–176)**

- **Design classification** – The study is described as a “retrospective follow‑up study” (lines 148–153). As data on initiation and discontinuation were collected at one time point using retrospective recall, the design is essentially a **retrospective cohort**. Clarify this in both abstract and methods and acknowledge the potential for recall bias when women report discontinuation dates.
- **Response:** We thank the reviewer for the crucial clarification. We have changed the study design to "retrospective cohort" in both the Abstract and Methods sections. In addition, we highlighted the possibility of recall bias in the Limitations section.
- **Study setting description** – The manuscript provides a detailed description of the refugee camps and explains why some camps were excluded for security reasons (lines 162–171). However, to assess generalisability, please provide basic demographic characteristics of the excluded camp or explain how refugees there may differ from those included. Explain why only Somali and South Sudanese camps were selected and whether cultural differences could influence discontinuation.
- **Response:** Thank you for your thoughtful feedback. We have included the description of the refugee camps under study settings. In addition, we explained that the excluded refugee camp included South Sudanese refugees with similar sociodemographic and cultural characteristics to those in the study (refugees from the Jewi camp). As a result, the exclusion is unlikely to alter the generalisability of our findings. We also indicated that Somali and Gambella refugee camps were selected because they had the highest refugee populations in Ethiopia, as already stated in the Study setting section. Although cultural differences may exist between these two groups, our analysis found that country of origin had no significant association with contraceptive discontinuation.

#### **Sample size and sampling (lines 182–223)**

- **Sample‑size calculation** – The sample size is calculated using a formula for survival analysis based on two proportions and a hazard ratio (lines 184–200). The formula and variables are not clearly formatted in the manuscript – the denominator of the formula and the source of the hazard ratio (HR = 1.74) are not explained. Please rewrite the formula in a readable format (e.g., as an equation) and justify the choice of HR = 1.74 and discontinuation probability of 18.4 % by citing the previous Congo study. As currently written, the derivation of 566 participants is difficult to follow.
- **Response**: Thank you for your important request to clarify the sample size calculation approaches. We have revised the sample-size calculation in the methods section (Page 8, lines 192-223) so that the formula and all variables are clearly displayed. In particular, the sample size equation shown in readable equation format, we clarified the choice of the hazard ratio from Casey eat al., 2017, and the reference citation included.
- **Sampling procedure** – All eligible women with complete records were included (lines 207–225), resulting in 551 participants. Provide counts for the women excluded due to incomplete records and specify whether excluded women differed from those included. Clarify how many camps were initially sampled and how the lottery method was implemented.
- **Response:** We appreciate the reviewer for this comment. We have clarified that **all eligible women with complete records were included,** resulting in 551 participants. A total of 8 women were excluded: 1 had hearing difficulties, 1 was sick at the time of the interview, and 6 had incomplete records. The excluded women had **similar demographic characteristics** to those included in the study.

Concerning the sampling of camps, out of 14 refugee camps (8 Somali and 6 South Sudanese), **3 Somali camps and 2 South Sudanese camps** were selected using a lottery method. Later, 1 South Sudanese camp (Tierkidi camp) was excluded for security reasons; the excluded camp hosted South Sudanese refugees with characteristics similar to those included, and thus its exclusion is unlikely to affect the representativeness of the study population. We have added these details to the Methods section to clarify eligibility, exclusions, and the sampling procedure (Page 9, lines 228-231 & 239-240).

#### **Data collection and quality assurance (lines 232–247, 281–291)**

- **Instrument development** – The questionnaire was adapted from existing literature and translated into local languages (lines 240–244). Include a brief summary of the main items (e.g., socio‑demographics, contraceptive history, partner variables) and refer to the supplementary file. Describe any pilot testing of the tool.
- **Response:** Thank you. A summary of the main items has been added, including **socio-demographic characteristics, Reproductive health history, and contraceptive history-related variables.** Detailed information on the questionnaire had already been provided in **S1_File (**supplementary material). Moreover, the questionnaire was pretested among 28 married women outside of the study camps to ensure clarity, relevance, and comprehension, as described in the Data Quality Control section.
- **Data quality** – The authors indicate that pre‑testing and extensive training were conducted and that data was collected electronically (lines 283–292). This is commendable. However, recall bias may still be present because discontinuation and switching dates were self‑reported. Acknowledge this limitation explicitly and consider cross‑checking with clinic records if possible.
- **Response:** Thank you for raising an important point. We have explicitly acknowledged **recall bias** as a limitation in the study. Discontinuation was assessed during the **first year of contraceptive use,** and most women were able to report the **exact dates** of discontinuation. To further ensure reliability, we **cross-checked the self-reported dates against FP records,** which were found to be similar.

#### **Variable definitions (lines 253–279)**

- **Outcome definitions** – The definitions of “continuer,” “switcher,” and “discontinuer” are clearly stated (lines 263–270). Nevertheless, this categorisation may misclassify women who temporarily discontinued and later restarted a method. Clarify whether women who switched to a different modern method were considered discontinuers or censored at the time of switch. Provide a rationale for limiting the discontinuation observation window to 12 months.
- **Response:** We appreciate the reviewer’s insightful comment. In our study, women who shifted to a different modern contraceptive method were considered discontinuers at the time of the shift. The observation period was limited to 12 months because discontinuation of contraceptives is most common during the first year of use, especially in the first three months following initiation. Early discontinuation is frequently caused by hormonal contraceptive side effects, issues with insertion or injection sites, misconceptions, and social pressure from peers, husbands, or family members. This one-year time period enables a more focused evaluation of early discontinuation patterns, which are crucial for understanding and enhancing contraceptive continuation in this segment of the population.
- **Exposures and covariates** – The manuscript lists socio‑demographic and reproductive variables but does not specify how variables like wealth index were derived (lines 256–259). Briefly describe the construction of the wealth index (e.g., principal component analysis on assets) and coding of categorical variables. For partner awareness and approval, specify the questions asked and the response options.
- **Response:** Thank you for raising this important comment. Regarding the wealth index, we used **self-declared wealth** and have now **consistently used the term “self-declared wealth”** throughout the manuscript. For partner awareness and approval, a substantial number of husbands/partners were not aware of or did not approve of their wives’ contraceptive use. To assess these variables, women were asked the following questions: **“Does your partner know about the method you have used/are using currently?”** and **“Does your partner approve of the method you have used/are using currently?”** The response options were “Yes” or “No.”

#### **Statistical analysis (lines 294–320)**

- **Candidate selection** – The authors used log‑rank tests to identify covariates with p < 0.25 for inclusion in the Cox model (lines 303–314). Relying solely on univariate p‑values may omit important confounders. Consider using conceptual frameworks or prior literature to select covariates. Variables such as education, parity and fertility desire are known predictors of discontinuation but were excluded because they lacked univariate significance.
- **Response**: Thank you for your comment. We believe that we did not rely solely on univariate p-values to select variables for the multivariable Cox model. Among the 12 candidate variables considered, some, such as **residence,** were included despite not being significant in the univariate analysis, based on their conceptual relevance and evidence from prior literature. Moreover, **parity and fertility intentions are indeed potential predictors for contraceptive continuation/discontinuation.** However, these were already captured through **women’s desire for more children and the number of children,** which were included in the analysis (Page 20, Table 5).
- **Proportional hazards assumption** – The methods state that proportional hazards (PH) assumptions were checked (lines 316–318) but no details are provided. Survival analysis literature emphasises that validating the PH assumption is crucial; graphical diagnostics and goodness‑of‑fit tests should be reported. Provide details on the tests used (e.g., Schoenfeld residuals, log‑minus‑log plots) and describe whether any variables violated the assumption. If violations were found, consider using stratified or time‑varying covariates.
- **Response:** Thank you for raising an important comment about the proportional hazard assumption. The proportional hazard assumption was checked using Schoenfeld residuals. Both global and covariate-specific tests were conducted. Covariates with p-values > 0.05 were considered to satisfy the PH assumption. We have updated the data processing and data analysis section under the methods part accordingly (Page 12, 339-345).
- **Handling of clustering and censoring** – Refugee women within camps may share unobserved characteristics. Describe whether clustering was assessed and whether robust standard errors were used. Clarify how censored observations (e.g., women still using contraception at 12 months) were handled.
- **Response:** Thank you so much for raising this point. We did not account for clustering by camp, as almost all refugee camps used similar family planning service delivery approaches. A similar organisation provided FP services following standardised guidelines and processes, reducing possible within-camp variability. However, we acknowledge that unmeasured similarities among camps may have contributed to some correlation between individuals' responses. Women who continued to use contraceptives after 12 months were categorised as right-censored, whereas those who switched to another contraceptive method were classified as discontinuers, consistent with previous method-specific discontinuation studies.

### 5. **Reviewer 2 comment (Results):**

#### **Presentation of descriptive results (lines 347–720)**

- **Tables and formatting** – Tables 1–4 are presented in plain text with extensive repetition of parentheses and percentages (lines 360–720). The formatting makes them difficult to read. Reformat tables to clearly list categories, counts and percentages with footnotes explaining abbreviations. Combine categories where appropriate (e.g., group age categories). Consider summarising key findings in the text and moving detailed tables to supplementary material.
- **Response**: Thank you for your valuable advice. We rounded all percentages and means to one decimal place in the Results and Tables. While we understand the importance of shortening the presentation, we believe it is clearer and more informative to show both numbers and percentages in the tables. The entire results section has been changed to improve clarity and minimize wordiness.
- **Interpretation of descriptive statistics** – The results section reports many comparisons between LARC and SAC users with p‑values but does not interpret their practical significance. For example, LARC users were more likely to have a longer mean stay in camp and to be urban residents (lines 360–420). Discuss why these differences might influence discontinuation rather than simply presenting p‑values.
- **Response:** We appreciate the reviewer's suggestions. While we recognise that interpreting the observed differences may add clarity, we opted to limit interpretation to the descriptive part to minimise overextension of the results section. Instead, major differences between LARC and SAC users, residence, and length of stay in camp were discussed in light of their potential influence on contraceptive discontinuation.

#### **Contraception dynamics (lines 720–840)**

- **Discontinuation rates** – The authors report discontinuation rates at three, six and twelve months and list reasons for discontinuation (lines 720–732). Present confidence intervals for these estimates. When comparing reasons for discontinuation between methods, use proportions rather than raw numbers to aid interpretation. Clarify how many women experienced method failure and whether pregnancy outcomes were recorded.
- **Response**: We appreciate the reviewer's comments. The reasons for contraceptive discontinuation were descriptively evaluated among women who discontinued a method (227 out of 551). Because several overlapping reasons were recorded, the numbers for each category were very small. To minimise confusion, we used actual numbers rather than proportions (Fig 3). However, as per the reviewers’ suggestion, we have removed the raw numbers and used proportions in the narratives. Furthermore, confidence intervals have already been added for the overall discontinuation rates at three, six, and twelve months. 47 women experienced method failure in their lifetime, with 9 of these happening while using the current/baseline method. When asked about the nine pregnancies, most individuals described them as "happy accidents."
- **Switching and abandonment** – Table 4 summarises switching and abandonment (lines 724–740). Clarify whether switching was considered a competing event or treated as censoring in the survival analysis. The term “abandonment” should be clearly defined earlier (lines 272–274) and its distinction from discontinuation explained.
- **Response:** Thank you. We have clarified about method switching above. In this study, abandonment is defined as “Termination of using any method altogether or discontinuation that is not followed by the restart of use at post-discontinuation.” The definitions for Method switching, abandonment, and method failure are presented in Study variable section

#### **Determinants of discontinuation (lines 840–960)**

- **Model output** – The Cox regression table (Table 5) provides crude and adjusted hazard ratios. Ensure that the reference categories are clearly stated. Note that some variables (e.g., wealth index, number of children) show attenuated associations in the adjusted model, suggesting confounding. The finding that urban residence and short camp stay are associated with higher discontinuation is interesting and warrants deeper exploration. Report model diagnostics (e.g., Akaike information criterion) and goodness‑of‑fit statistics.
- **Response**: We are grateful for the reviewer's feedback. Table 5 already includes the crude and adjusted hazard ratios with CIs and identifies reference categories as "1 (ref)", with a footnote noting that "Ref = Indicates the reference/the baseline category. " The attenuation of some associations (e.g., wealth index, number of children) likely reflects confounding by other socio-demographic factors. The associations of urban residence and shorter camp stay with higher discontinuation were further explored in the discussion. Model diagnostics, such as AIC and the global Schoenfeld residual test (p > 0.05), indicated good model fit and fulfillment with the proportional hazard assumption.
- **Interpretation of hazard ratios** – Use correct terminology when interpreting hazard ratios; they estimate instantaneous risk rather than absolute risk. For continuous variables like length of stay, specify the unit of increase (1 year). Acknowledge that the hazard ratio of 1.50 for SAC users indicates a 50 % higher instantaneous hazard compared with LARC users, but cumulative risk differences may differ over time.
- **Response**: Thank you for pointing this out. The interpretation about method type (SAC Vs LARC) and residence (rural vs. urban) is revised as per the suggestions.

### 6. **Reviewer 2 comment (Discussion and conclusion (lines 960–1089)):**

- **Comparison with literature** – The discussion compares the discontinuation rate to studies from Uganda, Congo and Myanmar (lines 960–975). Broaden the comparison to include recent DHS meta‑analyses or systematic reviews on discontinuation. When comparing rates, consider differences in population (married vs all women), recall periods and method mix. Avoid speculative explanations without evidence; for example, attributing higher discontinuation among refugees to “unstable life and relocation” (lines 990–1004) should be supported by data or qualitative insights.
- **Response:** We have revised the Discussion to include recent DHS analyses and meta-analyses (Page 22, lines 1904-1908). In addition, we added a qualitative study that identified living in the refugee camp was associated with restricted access to healthcare services (Luckenbill et al., 2025). Furthermore, we noted that differences in reported rates may reflect variations in population, recall period, and method mix.
- **Mechanisms and implications** – The manuscript hypothesises that SAC users discontinue because they can stop without visiting a clinic (lines 997–1002). Provide references to support this and discuss how counselling and follow‑up could mitigate discontinuation. The observation that urban residents have higher discontinuation is interesting; propose mechanisms (e.g., quality expectations, service interruptions) and consider alternative explanations. Discuss how partner occupation influences discontinuation and whether male engagement programmes might help.
- **Response:** Thank you for your feedback. We have added a reference citation that shows short-acting contraceptive users are more likely to stop contraceptive methods without healthcare providers’ assistance. Moreover, we have discussed how enhancing counseling services and continuous follow-up help to prevent early discontinuation. Furthermore, we discuss how partner occupation affects contraceptive use and how male involvement interventions might promote continuation through shared decision-making and open communication
- **Limitations** – The authors acknowledge exclusion of one camp and potential recall bias (lines 1074–1091). Additionally, discuss selection bias due to excluding women with incomplete records and the limited generalisability to unmarried women. Recognise the potential for misclassification of discontinuation episodes and for unmeasured confounding. Describe any steps taken to minimise these biases.
- **Response**: We appreciate the reviewer for providing this feedback. The limitation of the study is revised according to the reviewer’s suggestions. Specifically, our study is limited to married women and cannot be generalised to unmarried women. We discussed incomplete records, exclusion of one refugee camp, and steps taken to minimize potential limitations (Page 26-27, lines 2043-2063).
- **Conclusions and recommendations** – The conclusion reiterate the high discontinuation rate and recommends prioritising SAC users, urban residents and women with short camp stays (lines 1080–1088). Emphasise that interventions should include improved counselling, easy access to method switching and male partner engagement. Encourage further mixed‑methods research to understand the sociocultural drivers of discontinuation.
- **Response**: Thank you for raising an important point. We have updated the conclusion and recommendation section. Enhancement of contraceptive service provision, targeted counseling by involving partners, and a further mixed-methods study to explore socio-cultural determinants of method-specific contraceptive discontinuation after the first year and subsequent years of use are recommended.

## 7. **Reviewer 2 comment (Minor comments and editorial issues):**

1. **Grammar and style** – Several sentences contain typographical errors or awkward phrasing (e.g., “contraception initiations” instead of “contraceptive initiations” and “contraception discontinuation” instead of “contraceptive discontinuation”). Review the manuscript thoroughly for grammar, punctuation and tense consistency. Use present tense for general statements and past tense for study findings.

**Response**: We have thoroughly reviewed the manuscript and tried to correct grammar, tense, and typographic errors. We have replaced “contraception discontinuation” consistently with contraceptive discontinuation.”

2. **Formatting of equations** – The sample‑size formula and hazard ratio definitions should be presented using mathematical notation rather than embedded within text (lines 197–201). Use standard notation for logarithms (e.g., $\log$ ) and ensure parentheses are balanced.

**Response**: We appreciate the reviewer's suggestion. The sample-size formula and hazard ratio definitions have been rewritten to utilise proper mathematical expressions, such as standard logarithm notation (log) and balanced parentheses, to improve clarity.

3. **Acronyms and abbreviations** – Define all acronyms (LARC, SAC, FP, ANC, ODK) at first use and use them consistently thereafter. Avoid switching between “contraception” and “contraceptives” within the same sentence.

**Response**: Thank you for the suggestion. All acronyms are defined at first use and used consistently throughout the document

4. **Tables and figures** – Provide captions that explain the content and include units and footnotes explaining abbreviations. Ensure that figures are legible and include number at risk in Kaplan–Meier plots. Consider presenting the reasons for discontinuation as a stacked bar chart to facilitate comparison by method.

**Response**: Thank you for your comment. The caption of the tables and figures is already presented in the manuscript. Specifically, the caption for the figures is presented in the document, while the figures are submitted separately as per the journal’s recommendation. In addition, we have explained units and abbreviations as a footnote in the tables. The reasons for contraception discontinuation are presented in a stacked bar chart, making it easier to compare methods. However, when we included the number at risk for both LARC and SAC in the Kaplan-Meier plot, the picture became a little congested. Therefore, we opted to keep the original figure in the paper.

5. **References** – Update the reference list to include more recent literature (2022–2025) on contraceptive discontinuation, especially from humanitarian contexts. Ensure that all in‑text citations are properly numbered and correspond to the reference list. Check the format of the reference numbering (e.g., use square brackets consistently) and remove extraneous spaces or punctuation.

**Response**: We have added relevant references pertinent to our study and revised the numbering and bracket formatting to ensure consistency throughout the manuscript.

## 8. **Reviewer 2 comment (Strengths of the study):**

- **Novel setting** – Conducting a discontinuation study in refugee camps fills a critical gap in reproductive‑health research and highlights challenges faced by displaced women.
- **Large sample and comprehensive data collection** – Including all eligible women from registries and conducting computer‑assisted interviews minimizes selection bias and reduces data‑entry errors. The study collected detailed socio‑demographic, reproductive, and partner information.
- **Policy relevance** – Identifying modifiable factors such as method type, residence, and partner occupation provides actionable insights for humanitarian agencies. The recommendation to enhance counseling and consider partner involvement aligns with WHO guidance on family‑planning programs.
- **Response**: Thank you so much for acknowledging the strength of our study.

## 9. **Reviewer 2 comment (Summary and recommendations):**

This manuscript addresses an important issue and provides useful data on contraceptive discontinuation among refugee women. The high discontinuation rates underscore the need for targeted interventions in humanitarian settings. However, the manuscript requires substantial revision to improve clarity, methodological transparency and statistical rigour. Clarify the retrospective cohort design, provide a clear sample‑size derivation, report diagnostic checks for the proportional hazards assumption, and enhance discussion of findings relative to the literature. Improving tables and figures and correcting grammatical errors will also enhance readability. Addressing these issues will strengthen the manuscript’s contribution to the field and increase its suitability for publication.

- **Response:** We thank the reviewer for constructive feedback. We have used the retrospective cohort study design and provided a detailed sample‑size derivation. Diagnostic checks for the proportional hazard assumption have been conducted and reported. The discussion has been expanded to situate our findings relative to recent literature. Tables and figures have been improved for clarity, and grammatical errors have been corrected throughout the manuscript. We believe these revisions strengthen the methodological transparency, statistical rigour, and overall readability of the manuscript, enhancing its contribution to the field.

Again, thank you for allowing us to strengthen our manuscript with your valuable comments and queries. We have worked hard to incorporate your feedback and hope that these revisions persuade you to accept our submission.

Sincerely,

Andamlak Gizaw Alamdo

Corresponding Author

Department of Health Service Management, Health Promotion, Reproductive Health, and Nutrition, School of Public Health, Saint Paul's Hospital Millennium Medical College, Addis Ababa, Ethiopia

[gizandal@gmail.com/andamlak.gizaw@sphmmc.edu.et](mailto:gizandal@gmail.com/andamlak.gizaw@sphmmc.edu.et)

+251912038993
