# Supplementary material for: Early contraceptive discontinuation and associated factors among married women initiating long-acting and short-acting contraceptives in humanitarian settings in Ethiopia: A retrospective cohort study
Source: PLoS One. 2026 Mar 27;21(3):e0345855. doi: 10.1371/journal.pone.0345855 (PMC13028423; doi:10.1371/journal.pone.0345855)
Supplement: Attachment — Submitted filename: Response_to_Reviewers_auresp_2.docx [file pone.0345855.s007.docx]

# Rebuttal Letter

Academic Editor

*PLOS ONE*

February 16, 2026

Dear academic editor,

Thank you for inviting us to submit a revised draft of our manuscript entitled "Early contraceptive discontinuation and associated factors among married women initiating long-acting and short-acting contraceptives in humanitarian settings in Ethiopia: A retrospective cohort study" to *PLOS ONE.* We also value the time and energy you and the other reviewers invested in providing thoughtful feedback on how to strengthen our paper. We are therefore delighted to resubmit our article for further consideration. We have made the required modifications in light of your extensive comments and suggestions. We hope our edits and responses below satisfactorily address all the issues and concerns you and the reviewers have noted.

To facilitate your review of our revisions, the following is a point-by-point response to the questions and comments in a letter dated February 16, 2026

**ACADEMIC EDITOR’S COMMENTS**

First, we sincerely thank you for your valuable time and for providing such insightful comments and suggestions to improve our paper

1. **Academic editor’s comment:** Reviewer 1 recommends acceptance, reviewer 2 suggests an additional editing and check of statistical results. In this respect, regarding the model in table 5, it is true that there might be too many variables in the model. It would help to carry out a likelihood-ratio test of joint significance for the categorical variables (together with the simple test already reported for dichotomous variables) and a strategy of eliminating from the model variables that are very far from being significant (you could choose the p-value threshold  eg: 0.25 or 0.10 (or even 0.05). Removing variables might make the estimation more precise for the rest of variables. You can include the results of both models or just the final model describing the variables that have been removed from the model.

- **Response:** We thank the Editor for this constructive comment regarding the specification and overall complexity of the multivariable survival model presented in Table 5.

A combination of conceptual relevance, prior literature evidence, and statistical screening guided covariate selection for the Cox proportional hazards model. Twelve candidate variables were initially considered. Variables with a p-value < 0.25 in the log-rank test were retained for multivariable analysis, and selected variables (e.g., residence) were included irrespective of univariate statistical significance due to their conceptual importance and potential confounding role. Model fit and complexity were evaluated using the Akaike Information Criterion (AIC), with preference given to models with lower AIC values while accounting for model complexity. Variables that did not meaningfully contribute to model fit were excluded, resulting in a more parsimonious model with improved precision of the remaining hazard ratio estimates. The final Cox model showed good overall fit to the data (likelihood ratio χ² (21) = 235.52, p < 0.001; AIC = 2575.92).

We have revised the Methods section to clarify the model selection strategy

**REVIEWER 1 COMMENTS:**

We are deeply grateful to Reviewer 1 for the careful reading of our manuscript and for the valuable comments and suggestions, which have greatly improved the overall quality of the paper.

1. **Reviewer 1 comment (Results):** Thank you for your revisions. I have a last few very comments on clarification: Line 310-311: I’m guessing the (p<.001) refers to the difference in country of origin for LARC and SAC acceptors - probably better to move the p-value closer to the country of origin, i.e., before ‘they were Muslim by religion’.

- **Response:** Thank you for pointing this out. We have moved the p-value so that it directly follows the variable “country of origin,” clarifying that it refers to the difference in country of origin between LARC and SAC acceptors.

2. **Reviewer 1 comment (Results):** Line 312: please add ‘years’ if you are referring to 14.7 and 12.0 years as the length of stay in the camps. As written, the time period is unclear.

- **Response:** We thank the reviewer for the suggestion. We have added “years” after the mean lengths of stay to clarify the time period.

3. **Reviewer 1 comment (Results):** Table 1: would you please confirm that the p<.001 is correct for Occupation? The data don’t look that different, and I know you collapsed 2 categories into one so just want to double check this is correct.

- **Response:** We thank the reviewer for highlighting this. Upon revisiting Table 1, we realized that the p-value reported for the “Occupation” variable was not updated after merging the “Housewife” and “Not working” categories based on the reviewer’s earlier suggestion. After correcting the chi-square calculation with the merged categories, the p-value is now accurate and has been updated in Table 1. We apologize for this oversight and appreciate the reviewer’s careful attention.

4. **Reviewer 1 comment (Results):** Line 368-369: I’m a little confused about 15% of LARC users reporting discontinuation because of lack of/no access to services. Once they received the implant or IUD, they would need access to services to discontinue the method, so this doesn’t make much sense to me. (Lack of access does make a lot of sense for SAC.)

- **Response:** We thank the reviewer for this comment. Upon revisiting the data, we confirmed that 15.1% (8/53) of LARC users and 12.1% (21/174) of SAC users reported lack of or limited access to services as **one of the reasons for discontinuation.** We would like to clarify that the discontinuation variable allowed multiple responses; thus, these percentages reflect participants who cited lack of access among several possible reasons, rather than necessarily the primary reason. In the refugee context, where family planning services, particularly LARC, may be inconsistently available or interrupted, this likely reflects challenges in accessing follow-up care or timely removal. We have revised the manuscript to clarify both the multiple-response nature of the variable and the contextual interpretation.

5. **Reviewer 1 comment (Results):** Figure 3: would you please change the Figure to show to % rather than numbers? % would be more meaningful, and would match the data in the narrative.

- **Response:** Thank you for this suggestion. We have revised Figure 3 to display **percentages instead of raw numbers**, which aligns with the narrative and improves clarity.

6. **Reviewer 1 comment (Discussion):** Line 462-465: In this sentence, how do counseling services and satisfaction influence discontinuation (ie do they generally increase or decrease discontinuation – or is it not consistent

- **Response:** We thank the reviewer for this comment. We have revised the manuscript to clarify that lack of counseling services and dissatisfaction with family planning services are generally associated with **higher rates of LARC discontinuation**. This aligns with the findings of a systematic review and meta-analysis of 20 studies in SSA. The manuscript now explicitly reflects the direction of this association to avoid ambiguity.

**REVIEWER 2 COMMENTS**

I commend the authors for their careful and constructive response to the previous round of review. The revised manuscript demonstrates substantial improvement in methodological clarity, transparency of reporting, and coherence across sections. Key issues raised earlier particularly regarding study design classification, sample size justification, sampling procedures, interpretation of hazard ratios, and articulation of limitations have been addressed in a satisfactory manner.

Overall, the manuscript now presents a technically sound retrospective cohort analysis of contraceptive discontinuation among refugee women in Ethiopia. The analytical approach is appropriate for the research question, and the main conclusions are broadly supported by the data.

- **Response:** We truly appreciate the reviewer for providing a clear summary and acknowledging the significance of this study. We appreciate the recognition of our work addressing contraceptive discontinuation among refugee women, a population that is frequently underrepresented in reproductive health studies. The comments were well taken, and they helped us improve the manuscript's clarity, organisation, and interpretation.

Thank you once again for allowing us to improve our manuscript with your insightful comments and questions. We have carefully addressed your feedback and hope that our revisions meet your expectations and support the acceptance of our submission.

Sincerely,

Andamlak Gizaw Alamdo

Corresponding Author

Department of Health Service Management, Health Promotion, Reproductive Health, and Nutrition, School of Public Health, Saint Paul's Hospital Millennium Medical College, Addis Ababa, Ethiopia

[gizandal@gmail.com/andamlak.gizaw@sphmmc.edu.et](mailto:gizandal@gmail.com/andamlak.gizaw@sphmmc.edu.et)

+251912038993
