# Supplementary material for: Early contraceptive discontinuation and associated factors among married women initiating long-acting and short-acting contraceptives in humanitarian settings in Ethiopia: A retrospective cohort study
Source: PLoS One. 2026 Mar 27;21(3):e0345855. doi: 10.1371/journal.pone.0345855 (PMC13028423; doi:10.1371/journal.pone.0345855)
Supplement: Peer review history

# Peer review: Decision Letter 0

**DOI:** 10.1371/journal.pone.0345855.r001

**Authors:** José Antonio Ortega

6 Oct 2025

PONE-D-25-08308

Early contraceptive discontinuation and associated factors among married women initiating long-acting and short-acting contraceptives in humanitarian settings in Ethiopia:

A retrospective follow-up study

PLOS ONE

Dear Dr. Alamdo,

Thank you for submitting your manuscript to PLOS ONE. After careful consideration, we feel that it has merit but does not fully meet PLOS ONE’s publication criteria as it currently stands. Therefore, we invite you to submit a revised version of the manuscript that addresses the points raised during the review process.

Two experts have reviewed the manuscript noting the need to substantially revise it in terms of its placement on the literature, details on the setting and the methods, and a concern about the validity of the statistical analysis given the lack of testing of its assumptions. There are also many smaller suggestions.

One further consideration that should be explicitly mentioned is the wealth index. It is a misleading term. The wealth index in the context of fertility surveys is an index constructed based on ownership of some products and characteristics of the house, not a self-declared perception. In order to avoid misinterpretation I suggest using "Self-declared wealth" or something along these lines.

Please submit your revised manuscript by Nov 20 2025 11:59PM. If you will need more time than this to complete your revisions, please reply to this message or contact the journal office at plosone@plos.org . When you're ready to submit your revision, log on to https://www.editorialmanager.com/pone/ and select the 'Submissions Needing Revision' folder to locate your manuscript file.. When you're ready to submit your revision, log on to https://www.editorialmanager.com/pone/ and select the 'Submissions Needing Revision' folder to locate your manuscript file.

Please include the following items when submitting your revised manuscript:

- A rebuttal letter that responds to each point raised by the academic editor and reviewer(s). You should upload this letter as a separate file labeled 'Response to Reviewers'.
- A marked-up copy of your manuscript that highlights changes made to the original version. You should upload this as a separate file labeled 'Revised Manuscript with Track Changes'.
- An unmarked version of your revised paper without tracked changes. You should upload this as a separate file labeled 'Manuscript'.

If you would like to make changes to your financial disclosure, please include your updated statement in your cover letter. Guidelines for resubmitting your figure files are available below the reviewer comments at the end of this letter.

If applicable, we recommend that you deposit your laboratory protocols in protocols.io to enhance the reproducibility of your results. Protocols.io assigns your protocol its own identifier (DOI) so that it can be cited independently in the future. For instructions see: https://journals.plos.org/plosone/s/submission-guidelines#loc-laboratory-protocols . Additionally, PLOS ONE offers an option for publishing peer-reviewed Lab Protocol articles, which describe protocols hosted on protocols.io. Read more information on sharing protocols at . Additionally, PLOS ONE offers an option for publishing peer-reviewed Lab Protocol articles, which describe protocols hosted on protocols.io. Read more information on sharing protocols at https://plos.org/protocols?utm_medium=editorial-email&utm_source=authorletters&utm_campaign=protocols ..

We look forward to receiving your revised manuscript.

Kind regards,

José Antonio Ortega, Ph.D.

Academic Editor

PLOS ONE

Journal requirements:

When submitting your revision, we need you to address these additional requirements.

1. Please ensure that your manuscript meets PLOS ONE's style requirements, including those for file naming. The PLOS ONE style templates can be found at

https://journals.plos.org/plosone/s/file?id=wjVg/PLOSOne_formatting_sample_main_body.pdf and

https://journals.plos.org/plosone/s/file?id=ba62/PLOSOne_formatting_sample_title_authors_affiliations.pdf

2. Please include a complete copy of PLOS’ questionnaire on inclusivity in global research in your revised manuscript. Our policy for research in this area aims to improve transparency in the reporting of research performed outside of researchers’ own country or community. The policy applies to researchers who have travelled to a different country to conduct research, research with Indigenous populations or their lands, and research on cultural artefacts. The questionnaire can also be requested at the journal’s discretion for any other submissions, even if these conditions are not met. Please find more information on the policy and a link to download a blank copy of the questionnaire here: https://journals.plos.org/plosone/s/best-practices-in-research-reporting. Please upload a completed version of your questionnaire as Supporting Information when you resubmit your manuscript.

3. In the ethics statement in the Methods, you have specified that verbal consent was obtained. Please provide additional details regarding how this consent was documented and witnessed, and state whether this was approved by the IRB.

4. We note that the grant information you provided in the ‘Funding Information’ and ‘Financial Disclosure’ sections do not match.

When you resubmit, please ensure that you provide the correct grant numbers for the awards you received for your study in the ‘Funding Information’ section.

5. In the online submission form, you indicated that [The data underlying the results presented in the study are available from the corresponding author].

All PLOS journals now require all data underlying the findings described in their manuscript to be freely available to other researchers, either 1. In a public repository, 2. Within the manuscript itself, or 3. Uploaded as supplementary information.

This policy applies to all data except where public deposition would breach compliance with the protocol approved by your research ethics board. If your data cannot be made publicly available for ethical or legal reasons (e.g., public availability would compromise patient privacy), please explain your reasons on resubmission and your exemption request will be escalated for approval.

6. Your ethics statement should only appear in the Methods section of your manuscript. If your ethics statement is written in any section besides the Methods, please move it to the Methods section and delete it from any other section. Please ensure that your ethics statement is included in your manuscript, as the ethics statement entered into the online submission form will not be published alongside your manuscript.

7. If the reviewer comments include a recommendation to cite specific previously published works, please review and evaluate these publications to determine whether they are relevant and should be cited. There is no requirement to cite these works unless the editor has indicated otherwise.

Additional Editor Comments (if provided):

[Note: HTML markup is below. Please do not edit.]

Reviewers' comments:

Reviewer's Responses to Questions

**Comments to the Author**

1. Is the manuscript technically sound, and do the data support the conclusions?

The manuscript must describe a technically sound piece of scientific research with data that supports the conclusions. Experiments must have been conducted rigorously, with appropriate controls, replication, and sample sizes. The conclusions must be drawn appropriately based on the data presented.

Reviewer #1: Yes

Reviewer #2: Yes

**********

2. Has the statistical analysis been performed appropriately and rigorously?

Reviewer #1: I Don't Know

Reviewer #2: Yes

**********

3. Have the authors made all data underlying the findings in their manuscript fully available?

The PLOS Data policy requires authors to make all data underlying the findings described in their manuscript fully available without restriction, with rare exception (please refer to the Data Availability Statement in the manuscript PDF file). The data should be provided as part of the manuscript or its supporting information, or deposited to a public repository. For example, in addition to summary statistics, the data points behind means, medians and variance measures should be available. If there are restrictions on publicly sharing data—e.g. participant privacy or use of data from a third party—those must be specified.-->requires authors to make all data underlying the findings described in their manuscript fully available without restriction, with rare exception (please refer to the Data Availability Statement in the manuscript PDF file). The data should be provided as part of the manuscript or its supporting information, or deposited to a public repository. For example, in addition to summary statistics, the data points behind means, medians and variance measures should be available. If there are restrictions on publicly sharing data—e.g. participant privacy or use of data from a third party—those must be specified.

Reviewer #1: Yes

Reviewer #2: Yes

**********

4. Is the manuscript presented in an intelligible fashion and written in standard English?

PLOS ONE does not copyedit accepted manuscripts, so the language in submitted articles must be clear, correct, and unambiguous. Any typographical or grammatical errors should be corrected at revision, so please note any specific errors here.

Reviewer #1: Yes

Reviewer #2: Yes

**********

5. Review Comments to the Author

Please use the space provided to explain your answers to the questions above. You may also include additional comments for the author, including concerns about dual publication, research ethics, or publication ethics. (Please upload your review as an attachment if it exceeds 20,000 characters)

Reviewer #1: Thank you for this important study. It is good to see studies on contraceptive discontinuation in humanitarian settings as there are so few. I have a few comments – some of which are substantive, but others are recommendation to make this a bit easier to read.

Abstract: is there a reason who switch between RH and SRH in the Background? Seems like you could just use SRH consistently.

Line 65-68: I believe these sentences need references. “Women in humanitarian settings are often unable to find contraceptive methods when only a small number would desire to become pregnant” and “In addition, as a strategy for war and conflicts, women and girls are at increased risk of early and forced marriage, sexual and gender-based abuse, sexual exploitation, and transactional sex.

Lines 71-73: Also needs a reference “Moreover, contraceptive provision can reduce maternal mortality and morbidity from unwanted pregnancies and unsafe abortions and help to prevent sexually transmitted infections from spreading within the population”

Lines 93-97: please add references for some of these statements.

Line 103: Isn’t reference 13 a study on contraceptive discontinuation in a humanitarian settings?

Introduction: would you please add a brief description of the selected refugee camps? For example, urban vs rural? Population size? Are contraceptive services available at health facilities in the selected camps?

Line 131: is FP provided only to married women? Is there a reason to limit your study population to only married women?

Line 197: what do you mean by ‘resumption of use at post-discontinuation’? Do you mean people who terminated method use without switching?

Methods: could you say a bit more about how you approached selected women for an interview – particularly since women gave no prior consent to be contacted about their contraceptive use? Presumably, women could feel that the services at the health facility were not confidential if a stranger showed up looking for them to talk about their contraceptive use. You specifically mention in Line 237 that the majority of participants kept their contraceptive use secret. How did you avoid breaking that confidentiality and making sure the fact that you interviewed them did not reveal their contraceptive use to others?

Results: I recommend that you reduce most % and ages to one decimal point (eg, 29.9 rather than 29.93 or 93.3% instead of 93.29%) as you present a lot of data. I also think it would be more clear if you present the %s in the narrative and only show the N’s in the table. I would recommend reducing the wordiness in the Results section. For example, you could also adjust text like in line 271 to say ‘42.1% of LARC and 40.0% of SAC acceptors…’ rather than write out ‘over forty percent’ and ‘nearly forty percent’

Line 265: what does 14.74 or 12.04 refer to? the mean number of years?

Line 269: all of the refugee camps were not rural? If not, please describe the context of the selected camps in the Introduction.

Tables: please reduce all % to one decimal place. It would be to indicate somewhere in the table that the number in parentheses is the % and the other number is the N (or just add the % sign after all numbers that are percents – the table is difficult to read as is).

Tables: To reduce the size of the tables, any variable with only Yes/No responses, you could report only the Yes.

Table 1: I don’t think you need to report mean age at initiation and mean age now since everyone in the sample initiated FP within the previous 12-18 months. It doesn’t really make sense that the mean age now is 3 years older than the age at initiation when only 12-18 months (should) have passed between the two dates. Can you explain this (or better yet, keep only one of these two age variables)?

Table 1: what is the difference between ‘Housewife’ and ‘Not working’ if everyone is married? Should these be collapsed into one category?

Line 292 and Table 2: was the desire for more children measured as within 2 years of now (time of interview) or of when they started using the method?

Line 302: could you say how partner approval was different? (Eg, higher or lower among LARC vs SAC acceptors?)

Line 307 and Table 3: were all participants asked about intention to use contraception in the future or only those who had terminated their contraceptive use? If all, what does that mean for women who were currently using a method?

Line 323: what is the difference between ‘experiencing side effects’ and ‘fear of side effects’? Seems like those could be combined.

Figure 3: Seems like the Y-axis could have a maximum of 100 given that is the highest number of women who gave a particular reason. Is there a reason you did not present this as %? Since you have so much going on in this chart, I recommend only presenting LARC vs SAC users. Or switch it to a table (could go into Table 4).

Line 328-329: ‘Among women who discontinued the baseline methods a substantial proportion of 32 (60.38%) LARC and nearly one-third of 56 (32.18%) SAC acceptors abandoned the method’ What did the rest who discontinued the method do? I’m not sure I understand this sentence.

Line 330-331: I just want to clarify – the question about method failure was for all women since it’s reported as ‘in their lifetime’ and not the reason for the current method discontinuation?

Lines 358-360: I’m still unclear about asking women who currently use FP about their intention to use in the future. I don’t think it makes sense to include in your regression model. If they are currently using, doesn’t that mean they’re likely to use in the future? Questions about intention to use FP in the future are usually asked of women who are NOT currently using FP.

Table 5: I am concerned about the number of categories you have in your regression models. Your N is not very large, so having 4-category variables in here (wealth index, husband occupation, no. of children) seems like it would reduce the power of the analysis. For example, would Husband occupation be more powerful to compare Not working vs Employed? There are only 62 people in the ‘private employee’ category and 12 in ‘Other’.

Table 5: I recommend using short acting and long acting rather than short term and long term to be consistent with how you’ve referred to the methods throughout the paper. Same for Line 374.

Line 380: I am familiar with this Congo study - these weren’t refugees, but rather conflict-affected populations.

Discussion: I would recommend that you report fewer % from your data in this section. You’ve already reported them in the Results. For example, lines 404-406, I recommend deleting the numbers by method.

Discussion: since side effects was a common reason for discontinuation, it may be good to reflect on how improvements to contraceptive counseling and information provided could potentially improve continuation. You sort of bring up quality of service delivery in your conclusion but seems like you could address this in the Discussion.

Lines 393-400: You mention multiple reasons for higher discontinuation among refugee women in your sample – do you know if any of these are true? For example, was there a lot of movement among the refugee populations in the selected camps?

Line 417-420: I’m a little confused by the statement here which seems to indicate that continuation should be higher in urban settings? That’s not what you found though?

Line 435: Here you contradict what you say in Line 397. Here you say the refugees may be more stable, but in Line 397 you say they’re less stable?

Lines 443-444: this statement needs a reference OR you should say they ‘may have’ to soften it since it is your inference, not necessarily a fact.

Line 479-480: do you have data on contraceptive prevalence among refugee populations in Ethiopia? This would be useful information to present in the Introduction. In general, I think there have been a number of studies on refugees in Ethiopia – could you reference some SRH data on refugees in Ethiopia in the Introduction or Discussion?

Lines 486-488: I’m not sure what you mean by ‘providers should place more emphasis on’ those specific categories?

Reviewer #2: 1. Is the manuscript technically sound, and do the data support the conclusions?

o Response: Yes, but only with major revisions.

The study addresses an important gap by examining contraceptive discontinuation among refugee women and has a clearly defined outcome. A moderate sample size (551 women) and use of survival analysis provide useful evidence. However, the design is essentially a retrospective cohort based on self reported dates, so recall bias is likely. Only married women were sampled; unmarried women and one camp were excluded, which limits generalisation. Stratifying short acting methods (injectables vs pills) is needed because discontinuation varies across methods. Despite these issues, the reported discontinuation rates and identified predictors broadly support the conclusions, but caution is warranted in interpreting causality.

2. Has the statistical analysis been performed appropriately and rigorously?

o Response: No, statistical methods need improvement.

The authors used Chi square tests and a Cox proportional hazards model. However, they do not report diagnostics for the proportional hazard’s assumption. Survival analysis literature emphasises that validating this assumption is crucial and that extended models should be used if it is violated. The hazard ratio cannot always remain constant, and tests such as log-minus log plots or goodness of fit measures are recommended. Furthermore, covariates were selected based on univariate p values, which may omit theoretical confounders; conceptual frameworks should guide confounder selection. The authors should also clarify whether they handled time varying covariates or conducted sensitivity analyses.

3. Have the authors made all data underlying the findings fully available?

o Response: Unsure / needs clarification.

In the submission form, the authors checked “Yes – all data are fully available without restriction”, but they did not provide a data repository link or accession number. PLOS policy requires depositing the de identified dataset in a public repository or including it in the supplementary files. The manuscript currently lacks details of where the data can be accessed. The authors should supply a data availability statement with a functional DOI or explicit explanation if data sharing is restricted.

4. Is the manuscript presented in an intelligible fashion and written in standard English?

o Response: Partially.

The manuscript is generally understandable, but grammatical errors and formatting problems impede readability. Several sentences in the abstract and abbreviations (LARC, SAC) are not defined on first use.

5. Review comments to the author

o Design and sampling: Clearly state that this is a retrospective cohort study. Describe how the 551 participants were identified and why 15 were excluded. Explain the characteristics of excluded camps and acknowledge that findings may not generalise to unmarried women or other refugee settings.

o Measurement of outcomes/exposures: Provide more detail on how discontinuation episodes were defined and handled. Consider stratifying short acting methods (pills vs injectables) to reflect their different adherence requirements. Explain how partner awareness, partner approval and wealth index were measured.

o Statistical analysis: Report tests for the proportional hazards assumption and, if violated, consider stratified or time dependent Cox models. Do not rely solely on univariate p values for confounder selection; include variables such as education, parity and fertility desire even if not significant in univariate analyses. Interpret hazard ratios correctly and specify the time units for continuous variables.

o Presentation and discussion: Improve table and figure formatting. Compare the discontinuation rates to other studies, including recent DHS analyses, and discuss why refugee settings may have higher rates. Expand the discussion of reasons for discontinuation, linking them to global evidence on fear of side effects, partner opposition and service quality. Provide a flow diagram showing participant selection.

o Ethics and data availability: Clarify how participants’ privacy was protected during interviews and provide information on any support mechanisms for women experiencing partner violence. Provide a clear data availability statement with a public repository link or justify why data cannot be shared.

**********

6. PLOS authors have the option to publish the peer review history of their article (what does this mean? ). If published, this will include your full peer review and any attached files.). If published, this will include your full peer review and any attached files.

If you choose “no”, your identity will remain anonymous but your review may still be made public.

**Do you want your identity to be public for this peer review?** For information about this choice, including consent withdrawal, please see our For information about this choice, including consent withdrawal, please see our Privacy Policy .-->.

Reviewer #1: No

Reviewer #2: No

**********

[NOTE: If reviewer comments were submitted as an attachment file, they will be attached to this email and accessible via the submission site. Please log into your account, locate the manuscript record, and check for the action link "View Attachments". If this link does not appear, there are no attachment files.]

While revising your submission, please upload your figure files to the Preflight Analysis and Conversion Engine (PACE) digital diagnostic tool, https://pacev2.apexcovantage.com/ . PACE helps ensure that figures meet PLOS requirements. To use PACE, you must first register as a user. Registration is free. Then, login and navigate to the UPLOAD tab, where you will find detailed instructions on how to use the tool. If you encounter any issues or have any questions when using PACE, please email PLOS at . PACE helps ensure that figures meet PLOS requirements. To use PACE, you must first register as a user. Registration is free. Then, login and navigate to the UPLOAD tab, where you will find detailed instructions on how to use the tool. If you encounter any issues or have any questions when using PACE, please email PLOS at figures@plos.org . Please note that Supporting Information files do not need this step.. Please note that Supporting Information files do not need this step.

Attachment Submitted filename: Peer review comments.docx

# Author comment: Author response to Decision Letter 1

**DOI:** 10.1371/journal.pone.0345855.r002

11 Dec 2025

Rebuttal Letter

Academic Editor

PLOS ONE

November 18, 2025

Dear academic editor,

Thank you for inviting us to submit a revised draft of our manuscript entitled "Early contraceptive discontinuation and associated factors among married women initiating long-acting and short-acting contraceptives in humanitarian settings in Ethiopia: A retrospective follow-up study" to PLOS ONE. We also value the time and energy you and the other reviewers invested in providing thoughtful feedback on how to strengthen our paper. We are therefore delighted to resubmit our article for further consideration. We have made the required modifications in light of your extensive comments and suggestions. We hope our edits and responses below satisfactorily address all the issues and concerns you and the reviewers have noted.

To facilitate your review of our revisions, the following is a point-by-point response to the questions and comments in a letter dated November 18, 2025

ACADEMIC EDITOR’S COMMENTS

First, thank you so much for your precious time, insightful comments, and suggestions for improving our paper.

Academic editor’s comment: The manuscript needs substantial revision in terms of its placement in the literature, details on the setting and methods, and testing the assumptions of the statistical analysis.

Response: Thank you for your important feedback. We have substantially revised the manuscript to better align with the literature (page 4, lines 93-97), (Page 5-6), Page (22, 1966-1971), clarified the setting and methods (Page 7, lines 167-171), and included the required diagnostic checks for statistical assumptions (Page 12, 339-345).

Academic editor’s comment: The term “Wealth Index” is misleading; in fertility surveys, it refers to an index based on household assets rather than self-declared perception. Please consider using “Self-declared wealth” or similar

Response: Thank you for your insightful suggestion. We agree with the concern and have changed "Wealth Index" to "Self-declared wealth" throughout the manuscript to avoid confusion.

Academic editor’s comment: The funding information provided in the ‘Funding Information’ and ‘Financial Disclosure’ sections do not match.

Response: We have corrected and harmonized the grant information in both sections. The grant number for WHO HRP–University of Ghana is 2022/1230378.

Academic editor’s comment: Please provide more information on how verbal consent was documented and whether this was approved by the IRB.

Response: We have revised the Ethics statement (Methods, Ethical consideration part (Page 13, lines 374-379) to explain how verbal consent was documented. After reading the participant's consent statement, the interviewer signed a record confirming that consent was obtained. This procedure was approved by the SPHMMC IRB (Ref no: PM23/401/23/01/2024)

Academic editor’s comment: Please ensure that your data are available according to PLOS ONE’s data policy.

Response: We have updated the Data Availability statement. The data underlying our findings are now provided within the manuscript and its Supporting Information files, in accordance with PLOS ONE’s data policy.

Academic editor’s comment: Please ensure that the ethics statement appears only in the Methods section.

Response: Thank you for the clarification. We confirm that the Ethics Statement is already included exclusively in the Methods section of the manuscript, and it does not appear in any other section. No changes were required, but we have revised it to ensure full compliance with PLOS ONE’s requirements.

Academic editor’s comment: Please include the PLOS inclusivity questionnaire as Supporting Information.

Response: The completed questionnaire has been added as Supporting Information (S2 Checklist)

REVIEWER 1 COMMENTS:

We are deeply grateful to Reviewer 1 for the careful reading of our manuscript and for the valuable comments and suggestions, which have greatly improved the overall quality of the paper.

Reviewer 1 comment (Abstract): Abstract: Is there a reason you switch between RH and SRH in the Background? It seems you could just use SRH consistently.

Response: We thank the reviewer for pointing this out. We have now used the term “sexual and reproductive health (SRH)” consistently throughout the abstract and the rest of the manuscript to maintain clarity and uniformity.

Reviewer 1 comment (Introduction): Lines 65–68: I believe these sentences need references: “Women in humanitarian settings are often unable to find contraceptive methods when only a small number would desire to become pregnant” and “In addition, as a strategy for war and conflicts, women and girls are at increased risk of early and forced marriage, sexual and gender-based abuse, sexual exploitation, and transactional sex.”

Response: We appreciate your observation. We have now added appropriate references to support these statements. The revised version cites [Achola et al., 2024] and [Tanabe et al., 2017] to substantiate the claims regarding limited contraceptive access and the risks faced by women and girls in humanitarian settings.

Reviewer 1 comment (Introduction): Lines 71–73: Also needs a reference. “Moreover, contraceptive provision can reduce maternal mortality and morbidity from unwanted pregnancies and unsafe abortions and help to prevent sexually transmitted infections from spreading within the population.”

Response: Thank you for this important suggestion. We have now included relevant citations [Ali, M. M., Cleland, J. G., & Shah, I. H., 2012] supporting the evidence that contraceptive provision contributes to reductions in maternal mortality, morbidity, and sexually transmitted infections.

Reviewer 1 comment (Introduction): Lines 93–97: Please add references for some of these statements.

Response: We agree and have now added several references [Khalifa et al., 2022; Mekonnen & Wubneh, 2020; Sedgh et al., 2014] to strengthen these statements and ensure that all factual claims are properly supported.

Reviewer 1 comment (Introduction): Line 103: Isn’t reference 13 a study on contraceptive discontinuation in a humanitarian setting?

Response: We thank the reviewer for noting this. We agree that reference 13 found evidence on contraception discontinuation in humanitarian settings. We have revised the sentence and clarified that, apart from a study by Casey in DRC, there remains a critical evidence gap on contraceptive discontinuation, method switching, method failure, and contraceptive abandonment in the humanitarian context.

Reviewer 1 comment (Introduction): Introduction: Would you please add a brief description of the selected refugee camps? For example, urban vs. rural? Population size? Are contraceptive services available at health facilities in the selected camps?

Response: Thank you to the reviewer for making this thoughtful recommendation. We agree that providing background information on refugee camps improves readers' understanding of the study's context. As a result, we have added a brief description of the camps, including their location (urban/rural classification), population size, and availability of contraceptive services, under the "Study Setting" section (Page 7, lines 167-171), which we believe is a more appropriate place for such contextual and methodological details.

Reviewer 1 comment (Methods): Line 131: Is FP provided only to married women? Is there a reason to limit your study population to only married women?

Response: We appreciate the reviewer raising this point. In the updated manuscript, we clarify the rationale for focusing on married women. In the study setting, the majority of FP clients at the RRS health center in selected refugee camps were married women, and the health facility registries primarily document information for married women. Furthermore, the sociocultural norms in these refugee camps often associate the use of contraceptives with marriage. As a result, we limited the study population to married women to ensure data accuracy and consistency with current healthcare data. We have included this explanation in the Methods section (Study participants and selection procedure) (Page 8, lines 182-188).

Reviewer 1 comment (Methods): Line 197: What do you mean by “resumption of use at post-discontinuation”? Do you mean people who terminated method use without switching?

Response: Thank you for your observations. The term "resumption of use at post-discontinuation" refers to women who stopped using a contraceptive method and then resumed using the same or a different method after a time of nonuse. To ensure clarity, we rephrased the sentence in the updated version (Methods, Study variable subsection) (Page 11, lines 297-298).

Reviewer 1 comment (Methods): Methods: Could you say a bit more about how you approached selected women for an interview, particularly since women gave no prior consent to be contacted about their contraceptive use? Presumably, women could feel that the services at the health facility were not confidential if a stranger showed up looking for them to talk about their contraceptive use. You specifically mention in Line 237 that the majority of participants kept their contraceptive use secret. How did you avoid breaking that confidentiality and making sure the fact that you interviewed them did not reveal their contraceptive use to others?

Response: Thank you for your important comment. We have added an explanation in the amended Methods section that states, "Health professionals who were already known to the women first informed eligible participants about the study and sought their verbal consent for the research team to approach them."

As stated in the sampling technique, initial contact was made exclusively through health workers known to the women, interviews were held in the participants' homes, and no identifying information was collected. The IRB examined and approved the approach, as well as the use of verbal informed consent due to the sensitive nature of the issue.

Reviewer 1 comment (Results): I recommend that you reduce most % and ages to one decimal point (eg, 29.9 rather than 29.93 or 93.3% instead of 93.29%) as you present a lot of data. I also think it would be more clear if you present the %s in the narrative and only show the N’s in the table. I would recommend reducing the wordiness in the Results section. For example, you could also adjust text like in line 271 to say ‘42.1% of LARC and 40.0% of SAC acceptors…’ rather than write out ‘over forty percent’ and ‘nearly forty percent’.

Response: Thank you for your valuable advice. We agree and rounded all percentages and means to one decimal place in the Results and Tables. While we understand the importance of shortening the presentation, we believe it is clearer and more informative to show both numbers and percentages in the tables. Furthermore, the Results section has been substantially changed to improve clarity and minimize wordiness

Reviewer 1 comment (Results): Line 265: what does 14.74 or 12.04 refer to? the mean number of years?

Response: Thank you for your comment. The numbers (14.74 and 12.04) represent the average length of stay in the refugee camp for LARC and SAC acceptors, as described in the text.

Reviewer 1 comment (Results): Line 269: all of the refugee camps were not rural? If not, please describe the context of the selected camps in the Introduction.

Response: Thank you for your observation. As per your prior recommendation, the location and context of the refugee camps have already been included in the study setting section under Methods (Page 7, lines 167-171).

Reviewer 1 comment (Results): Tables: please reduce all % to one decimal place. It would be good to indicate somewhere in the table that the number in parentheses is the % and the other number is the N.

Response: Done. Percentages in tables have been revised to one decimal point. We also indicated values with percentages in the table headings.

Reviewer 1 comment (Results): Tables: To reduce the size of the tables, any variable with only Yes/No responses, you could report only the Yes.

Response: Thank you for your suggestion. We have updated the tables to include only the "Yes" category for binary variables.

Reviewer 1 comment (Results): Table 1: I don’t think you need to report mean age at initiation and mean age now since everyone in the sample initiated FP within the previous 12–18 months…

Response: Thank you for this comment. The woman's age at contraceptive initiation refers to the first time she used any contraceptive method in her life, whereas the respondent’s age is her age at the time of the interview. To capture this distinction, we kept both variables.

Reviewer 1 comment (Results): Table 1: what is the difference between ‘Housewife’ and ‘Not working’ if everyone is married? Should these be collapsed?

Response: Thank you for noticing this. To improve clarity, we combined these two categories into a single variable ("Not working/Housewife").

Reviewer 1 comment (Results): Line 292 and Table 2: was the desire for more children measured as within 2 years of now or of when they started using the method?

Response: The desire for more children was assessed in relation to the timing of the interview. We clarified this in the paper as a footnote

Reviewer 1 comment (Results): Line 302: could you say how partner approval was different?

Response: Yes, thank you. We now describe that LARC users had higher partner approval than SAC users.

Reviewer 1 comment (Results): Line 307 and Table 3: were all participants asked about intention to use contraception in the future or only those who had terminated their contraceptive use?

Response: Thank you. The question on future intention to use contraception was asked of all women to capture both potential use among non-users and continuation intentions among current users, using language that clearly enabled women to indicate whether they intended to continue, switch, or discontinue using contraception.

Reviewer 1 comment (Results): Line 323: what is the difference between ‘experiencing side effects’ and ‘fear of side effects’? Seems like those could be combined.

Response: Thank you for your comment. We maintained the terms 'fear of side effects' and 'experiencing side effects' separate since fear of side effects is usually linked to misconceptions or peer pressure influencing non-use or discontinuation, whereas experiencing side effects refers to actual method-related complaints that vary among users. Distinguishing these two provides valuable evidence for developing targeted interventions.

Reviewer 1 comment (Results): Figure 3: Seems like the Y-axis could have a maximum of 100…

Response: Thank you for your comment. The reasons for contraception discontinuation are presented in a stacked bar chart, making it easier to compare methods.

Reviewer 1 comment (Results): Line 328–329: ‘Among women who discontinued…’ What did the rest who discontinued the method do?

Response: Thank you for your question. A total of 227 women discontinued their baseline method. Of these, 88 stopped using contraception entirely, indicating that they had no plans to use any kind of method again. The remaining women either switched to another method or temporarily discontinued for reasons such as planning to become pregnant or having become pregnant, to resume or restart contraception later, either the same or a different method.

Reviewer 1 comment (Results): Line 330–331: clarify method failure question.

Response: Thank you for your comment. Because of the small number of women who encountered method failure/got pregnant while using contraceptives/, the method failure question in this study focuses on lifetime experience rather than the reason for current discontinuation. Out of 551 participants, 47 women reported method failure in their lifetime. Of those who experienced method failure (47), 9 women reported while using the baseline method. Method failure is explicitly defined under the study variables in the methods section.

Reviewer 1 comment (Results): Lines 358–360: intention to use FP for current users.

Response: This has already been explained above.

Reviewer 1 c

Attachment Submitted filename: Response to Reviewers.docx

# Peer review: Decision Letter 1

**DOI:** 10.1371/journal.pone.0345855.r003

**Authors:** José Antonio Ortega

1 Feb 2026

PONE-D-25-08308R1

Early contraceptive discontinuation and associated factors among married women initiating long-acting and short-acting contraceptives in humanitarian settings in Ethiopia: A retrospective cohort study

PLOS One

Dear Dr. Alamdo,

Thank you for submitting your manuscript to PLOS ONE. After careful consideration, we feel that it has merit but does not fully meet PLOS ONE’s publication criteria as it currently stands. Therefore, we invite you to submit a revised version of the manuscript that addresses the points raised during the review process.

Both reviewers were available. Reviewer 1 recommends acceptance, reviewer 2 suggests an additional editing and check of statistical results. In this respect, regarding the model in table 5, it is true that there might be too many variables in the model. It would help to carry out a likelihood-ratio test of joint significance for the categorical variables (together with the simple test already reported for dichotomous variables) and a strategy of eliminating from the model variables that are very far from being significant (you could choose the p-value threshold  eg: 0.25 or 0.10 (or even 0.05). Removing variables might make the estimation more precise for the rest of variables. You can include the results of both models or just the final model describing the variables that have been removed from the model.

Please submit your revised manuscript by Mar 18 2026 11:59PM. If you will need more time than this to complete your revisions, please reply to this message or contact the journal office at plosone@plos.org . When you're ready to submit your revision, log on to https://www.editorialmanager.com/pone/ and select the 'Submissions Needing Revision' folder to locate your manuscript file.. When you're ready to submit your revision, log on to https://www.editorialmanager.com/pone/ and select the 'Submissions Needing Revision' folder to locate your manuscript file.

Please include the following items when submitting your revised manuscript:

- A letter that responds to each point raised by the academic editor and reviewer(s). You should upload this letter as a separate file labeled 'Response to Reviewers'.
- A marked-up copy of your manuscript that highlights changes made to the original version. You should upload this as a separate file labeled 'Revised Manuscript with Track Changes'.
- An unmarked version of your revised paper without tracked changes. You should upload this as a separate file labeled 'Manuscript'.

-->If you would like to make changes to your financial disclosure, please include your updated statement in your cover letter. Guidelines for resubmitting your figure files are available below the reviewer comments at the end of this letter.

If applicable, we recommend that you deposit your laboratory protocols in protocols.io to enhance the reproducibility of your results. Protocols.io assigns your protocol its own identifier (DOI) so that it can be cited independently in the future. For instructions see: https://journals.plos.org/plosone/s/submission-guidelines#loc-laboratory-protocols . Additionally, PLOS ONE offers an option for publishing peer-reviewed Lab Protocol articles, which describe protocols hosted on protocols.io. Read more information on sharing protocols at . Additionally, PLOS ONE offers an option for publishing peer-reviewed Lab Protocol articles, which describe protocols hosted on protocols.io. Read more information on sharing protocols at https://plos.org/protocols?utm_medium=editorial-email&utm_source=authorletters&utm_campaign=protocols ..

We look forward to receiving your revised manuscript.

Kind regards,

José Antonio Ortega, Ph.D.

Academic Editor

PLOS One

Journal Requirements:

If the reviewer comments include a recommendation to cite specific previously published works, please review and evaluate these publications to determine whether they are relevant and should be cited. There is no requirement to cite these works unless the editor has indicated otherwise.

Please review your reference list to ensure that it is complete and correct. If you have cited papers that have been retracted, please include the rationale for doing so in the manuscript text, or remove these references and replace them with relevant current references. Any changes to the reference list should be mentioned in the rebuttal letter that accompanies your revised manuscript. If you need to cite a retracted article, indicate the article’s retracted status in the References list and also include a citation and full reference for the retraction notice.

[Note: HTML markup is below. Please do not edit.]

Reviewers' comments:

Reviewer's Responses to Questions

**Comments to the Author**

1. If the authors have adequately addressed your comments raised in a previous round of review and you feel that this manuscript is now acceptable for publication, you may indicate that here to bypass the “Comments to the Author” section, enter your conflict of interest statement in the “Confidential to Editor” section, and submit your "Accept" recommendation.

Reviewer #1: (No Response)

Reviewer #2: All comments have been addressed

**********

2. Is the manuscript technically sound, and do the data support the conclusions?

The manuscript must describe a technically sound piece of scientific research with data that supports the conclusions. Experiments must have been conducted rigorously, with appropriate controls, replication, and sample sizes. The conclusions must be drawn appropriately based on the data presented.

Reviewer #1: Yes

Reviewer #2: Yes

**********

3. Has the statistical analysis been performed appropriately and rigorously?

Reviewer #1: I Don't Know

Reviewer #2: Yes

**********

4. Have the authors made all data underlying the findings in their manuscript fully available?

The PLOS Data policy requires authors to make all data underlying the findings described in their manuscript fully available without restriction, with rare exception (please refer to the Data Availability Statement in the manuscript PDF file). The data should be provided as part of the manuscript or its supporting information, or deposited to a public repository. For example, in addition to summary statistics, the data points behind means, medians and variance measures should be available. If there are restrictions on publicly sharing data—e.g. participant privacy or use of data from a third party—those must be specified.-->requires authors to make all data underlying the findings described in their manuscript fully available without restriction, with rare exception (please refer to the Data Availability Statement in the manuscript PDF file). The data should be provided as part of the manuscript or its supporting information, or deposited to a public repository. For example, in addition to summary statistics, the data points behind means, medians and variance measures should be available. If there are restrictions on publicly sharing data—e.g. participant privacy or use of data from a third party—those must be specified.

Reviewer #1: Yes

Reviewer #2: Yes

**********

5. Is the manuscript presented in an intelligible fashion and written in standard English?

PLOS ONE does not copyedit accepted manuscripts, so the language in submitted articles must be clear, correct, and unambiguous. Any typographical or grammatical errors should be corrected at revision, so please note any specific errors here.

Reviewer #1: Yes

Reviewer #2: Yes

**********

6. Review Comments to the Author

Please use the space provided to explain your answers to the questions above. You may also include additional comments for the author, including concerns about dual publication, research ethics, or publication ethics. (Please upload your review as an attachment if it exceeds 20,000 characters)

Reviewer #1: Thank you for your revisions. I have a last few very comments on clarification:

Line 310-311: I’m guessing the (p<.001) refers to the difference in country of origin for LARC and SAC acceptors - probably better to move the p-value closer to the country of origin, ie before ‘they were Muslim by religion’.

Line 312: please add ‘years’ if you are referring to 14.7 and 12.0 years as the length of stay in the camps. As written, the time period is unclear.

Table 1: would you please confirm that the p<.001 is correct for Occupation? The data don’t look that different, and I know you collapsed 2 categories into one so just want to double check this is correct.

Line 368-369: I’m a little confused about 15% of LARC users reporting discontinuation because of lack of/no access to services. Once they received the implant or IUD, they would need access to services to discontinue the method, so this doesn’t make much sense to me. (Lack of access does make a lot of sense for SAC.)

Figure 3: would you please change the Figure to show to % rather than numbers? % would be more meaningful, and would match the data in the narrative.

Line 462-465: In this sentence, how do counseling services and satisfaction influence discontinuation (ie do they generally increase or decrease discontinuation – or is it not consistent)?

Reviewer #2: I commend the authors for their careful and constructive response to the previous round of review. The revised manuscript demonstrates substantial improvement in methodological clarity, transparency of reporting, and coherence across sections. Key issues raised earlier particularly regarding study design classification, sample size justification, sampling procedures, interpretation of hazard ratios, and articulation of limitations have been addressed in a satisfactory manner.

Overall, the manuscript now presents a technically sound retrospective cohort analysis of contraceptive discontinuation among refugee women in Ethiopia. The analytical approach is appropriate for the research question, and the main conclusions are broadly supported by the data.

**********

7. PLOS authors have the option to publish the peer review history of their article (what does this mean? ). If published, this will include your full peer review and any attached files.). If published, this will include your full peer review and any attached files.

If you choose “no”, your identity will remain anonymous but your review may still be made public.

**Do you want your identity to be public for this peer review?** For information about this choice, including consent withdrawal, please see our For information about this choice, including consent withdrawal, please see our Privacy Policy .-->.

Reviewer #1: No

Reviewer #2: No

**********

[NOTE: If reviewer comments were submitted as an attachment file, they will be attached to this email and accessible via the submission site. Please log into your account, locate the manuscript record, and check for the action link "View Attachments". If this link does not appear, there are no attachment files.]

To ensure your figures meet our technical requirements, please review our figure guidelines: https://journals.plos.org/plosone/s/figures

You may also use PLOS’s free figure tool, NAAS, to help you prepare publication quality figures: https://journals.plos.org/plosone/s/figures#loc-tools-for-figure-preparation.

NAAS will assess whether your figures meet our technical requirements by comparing each figure against our figure specifications.

# Author comment: Author response to Decision Letter 2

**DOI:** 10.1371/journal.pone.0345855.r004

16 Feb 2026

Academic Editor

PLOS ONE

February 16, 2026

Dear academic editor,

Thank you for inviting us to submit a revised draft of our manuscript entitled "Early contraceptive discontinuation and associated factors among married women initiating long-acting and short-acting contraceptives in humanitarian settings in Ethiopia: A retrospective cohort study" to PLOS ONE. We also value the time and energy you and the other reviewers invested in providing thoughtful feedback on how to strengthen our paper. We are therefore delighted to resubmit our article for further consideration. We have made the required modifications in light of your extensive comments and suggestions. We hope our edits and responses below satisfactorily address all the issues and concerns you and the reviewers have noted.

To facilitate your review of our revisions, the following is a point-by-point response to the questions and comments in a letter dated February 16, 2026

ACADEMIC EDITOR’S COMMENTS

First, we sincerely thank you for your valuable time and for providing such insightful comments and suggestions to improve our paper

1. Academic editor’s comment: Reviewer 1 recommends acceptance, reviewer 2 suggests an additional editing and check of statistical results. In this respect, regarding the model in table 5, it is true that there might be too many variables in the model. It would help to carry out a likelihood-ratio test of joint significance for the categorical variables (together with the simple test already reported for dichotomous variables) and a strategy of eliminating from the model variables that are very far from being significant (you could choose the p-value threshold eg: 0.25 or 0.10 (or even 0.05). Removing variables might make the estimation more precise for the rest of variables. You can include the results of both models or just the final model describing the variables that have been removed from the model.

Response: We thank the Editor for this constructive comment regarding the specification and overall complexity of the multivariable survival model presented in Table 5.

A combination of conceptual relevance, prior literature evidence, and statistical screening guided covariate selection for the Cox proportional hazards model. Twelve candidate variables were initially considered. Variables with a p-value < 0.25 in the log-rank test were retained for multivariable analysis, and selected variables (e.g., residence) were included irrespective of univariate statistical significance due to their conceptual importance and potential confounding role. Model fit and complexity were evaluated using the Akaike Information Criterion (AIC), with preference given to models with lower AIC values while accounting for model complexity. Variables that did not meaningfully contribute to model fit were excluded, resulting in a more parsimonious model with improved precision of the remaining hazard ratio estimates. The final Cox model showed good overall fit to the data (likelihood ratio χ² (21) = 235.52, p < 0.001; AIC = 2575.92).

We have revised the Methods section to clarify the model selection strategy

REVIEWER 1 COMMENTS:

We are deeply grateful to Reviewer 1 for the careful reading of our manuscript and for the valuable comments and suggestions, which have greatly improved the overall quality of the paper.

1. Reviewer 1 comment (Results): Thank you for your revisions. I have a last few very comments on clarification: Line 310-311: I’m guessing the (p<.001) refers to the difference in country of origin for LARC and SAC acceptors - probably better to move the p-value closer to the country of origin, i.e., before ‘they were Muslim by religion’.

Response: Thank you for pointing this out. We have moved the p-value so that it directly follows the variable “country of origin,” clarifying that it refers to the difference in country of origin between LARC and SAC acceptors.

2. Reviewer 1 comment (Results): Line 312: please add ‘years’ if you are referring to 14.7 and 12.0 years as the length of stay in the camps. As written, the time period is unclear.

Response: We thank the reviewer for the suggestion. We have added “years” after the mean lengths of stay to clarify the time period.

3. Reviewer 1 comment (Results): Table 1: would you please confirm that the p<.001 is correct for Occupation? The data don’t look that different, and I know you collapsed 2 categories into one so just want to double check this is correct.

Response: We thank the reviewer for highlighting this. Upon revisiting Table 1, we realized that the p-value reported for the “Occupation” variable was not updated after merging the “Housewife” and “Not working” categories based on the reviewer’s earlier suggestion. After correcting the chi-square calculation with the merged categories, the p-value is now accurate and has been updated in Table 1. We apologize for this oversight and appreciate the reviewer’s careful attention.

4. Reviewer 1 comment (Results): Line 368-369: I’m a little confused about 15% of LARC users reporting discontinuation because of lack of/no access to services. Once they received the implant or IUD, they would need access to services to discontinue the method, so this doesn’t make much sense to me. (Lack of access does make a lot of sense for SAC.)

Response: We thank the reviewer for this comment. Upon revisiting the data, we confirmed that 15.1% (8/53) of LARC users and 12.1% (21/174) of SAC users reported lack of or limited access to services as one of the reasons for discontinuation. We would like to clarify that the discontinuation variable allowed multiple responses; thus, these percentages reflect participants who cited lack of access among several possible reasons, rather than necessarily the primary reason. In the refugee context, where family planning services, particularly LARC, may be inconsistently available or interrupted, this likely reflects challenges in accessing follow-up care or timely removal. We have revised the manuscript to clarify both the multiple-response nature of the variable and the contextual interpretation.

5. Reviewer 1 comment (Results): Figure 3: would you please change the Figure to show to % rather than numbers? % would be more meaningful, and would match the data in the narrative.

Response: Thank you for this suggestion. We have revised Figure 3 to display percentages instead of raw numbers, which aligns with the narrative and improves clarity.

6. Reviewer 1 comment (Discussion): Line 462-465: In this sentence, how do counseling services and satisfaction influence discontinuation (ie do they generally increase or decrease discontinuation – or is it not consistent

Response: We thank the reviewer for this comment. We have revised the manuscript to clarify that lack of counseling services and dissatisfaction with family planning services are generally associated with higher rates of LARC discontinuation. This aligns with the findings of a systematic review and meta-analysis of 20 studies in SSA. The manuscript now explicitly reflects the direction of this association to avoid ambiguity.

REVIEWER 2 COMMENTS

I commend the authors for their careful and constructive response to the previous round of review. The revised manuscript demonstrates substantial improvement in methodological clarity, transparency of reporting, and coherence across sections. Key issues raised earlier particularly regarding study design classification, sample size justification, sampling procedures, interpretation of hazard ratios, and articulation of limitations have been addressed in a satisfactory manner.

Overall, the manuscript now presents a technically sound retrospective cohort analysis of contraceptive discontinuation among refugee women in Ethiopia. The analytical approach is appropriate for the research question, and the main conclusions are broadly supported by the data.

Response: We truly appreciate the reviewer for providing a clear summary and acknowledging the significance of this study. We appreciate the recognition of our work addressing contraceptive discontinuation among refugee women, a population that is frequently underrepresented in reproductive health studies. The comments were well taken, and they helped us improve the manuscript's clarity, organisation, and interpretation.

Thank you once again for allowing us to improve our manuscript with your insightful comments and questions. We have carefully addressed your feedback and hope that our revisions meet your expectations and support the acceptance of our submission.

Sincerely,

Andamlak Gizaw Alamdo

Corresponding Author

Department of Health Service Management, Health Promotion, Reproductive Health, and Nutrition, School of Public Health, Saint Paul's Hospital Millennium Medical College, Addis Ababa, Ethiopia

gizandal@gmail.com/andamlak.gizaw@sphmmc.edu.et

+251912038993

Attachment Submitted filename: Response_to_Reviewers_auresp_2.docx

# Peer review: Decision Letter 2

**DOI:** 10.1371/journal.pone.0345855.r005

**Authors:** José Antonio Ortega

11 Mar 2026

<p>Early contraceptive discontinuation and associated factors among married women initiating long-acting and short-acting contraceptives in humanitarian settings in Ethiopia: A retrospective cohort study

PONE-D-25-08308R2

Dear Dr. Alamdo,

We’re pleased to inform you that your manuscript has been judged scientifically suitable for publication and will be formally accepted for publication once it meets all outstanding technical requirements.

Within one week, you’ll receive an e-mail detailing the required amendments. When these have been addressed, you’ll receive a formal acceptance letter and your manuscript will be scheduled for publication.

An invoice will be generated when your article is formally accepted. Please note, if your institution has a publishing partnership with PLOS and your article meets the relevant criteria, all or part of your publication costs will be covered. Please make sure your user information is up-to-date by logging into Editorial Manager at Editorial Manager®  and clicking the ‘Update My Information' link at the top of the page. For questions related to billing, please contact  and clicking the ‘Update My Information' link at the top of the page. For questions related to billing, please contact billing support ..

If your institution or institutions have a press office, please notify them about your upcoming paper to help maximize its impact. If they’ll be preparing press materials, please inform our press team as soon as possible -- no later than 48 hours after receiving the formal acceptance. Your manuscript will remain under strict press embargo until 2 pm Eastern Time on the date of publication. For more information, please contact onepress@plos.org.

Kind regards,

José Antonio Ortega, Ph.D.

Academic Editor

PLOS One

Additional Editor Comments (optional):

One of the reviewers recommended acceptance on the previous draft. The remaining reviewer is satisfied with the change implemented to address their comments on the last version. The manuscript is ready for publication,

Reviewers' comments:

Reviewer's Responses to Questions

**Comments to the Author**

1. If the authors have adequately addressed your comments raised in a previous round of review and you feel that this manuscript is now acceptable for publication, you may indicate that here to bypass the “Comments to the Author” section, enter your conflict of interest statement in the “Confidential to Editor” section, and submit your "Accept" recommendation.

Reviewer #1: All comments have been addressed

**********

2. Is the manuscript technically sound, and do the data support the conclusions?

The manuscript must describe a technically sound piece of scientific research with data that supports the conclusions. Experiments must have been conducted rigorously, with appropriate controls, replication, and sample sizes. The conclusions must be drawn appropriately based on the data presented.

Reviewer #1: Yes

**********

3. Has the statistical analysis been performed appropriately and rigorously?

Reviewer #1: I Don't Know

**********

4. Have the authors made all data underlying the findings in their manuscript fully available?

The PLOS Data policy requires authors to make all data underlying the findings described in their manuscript fully available without restriction, with rare exception (please refer to the Data Availability Statement in the manuscript PDF file). The data should be provided as part of the manuscript or its supporting information, or deposited to a public repository. For example, in addition to summary statistics, the data points behind means, medians and variance measures should be available. If there are restrictions on publicly sharing data—e.g. participant privacy or use of data from a third party—those must be specified.-->requires authors to make all data underlying the findings described in their manuscript fully available without restriction, with rare exception (please refer to the Data Availability Statement in the manuscript PDF file). The data should be provided as part of the manuscript or its supporting information, or deposited to a public repository. For example, in addition to summary statistics, the data points behind means, medians and variance measures should be available. If there are restrictions on publicly sharing data—e.g. participant privacy or use of data from a third party—those must be specified.

Reviewer #1: Yes

**********

5. Is the manuscript presented in an intelligible fashion and written in standard English?

PLOS ONE does not copyedit accepted manuscripts, so the language in submitted articles must be clear, correct, and unambiguous. Any typographical or grammatical errors should be corrected at revision, so please note any specific errors here.

Reviewer #1: Yes

**********

6. Review Comments to the Author

Please use the space provided to explain your answers to the questions above. You may also include additional comments for the author, including concerns about dual publication, research ethics, or publication ethics. (Please upload your review as an attachment if it exceeds 20,000 characters)

Reviewer #1: (No Response)

**********

7. PLOS authors have the option to publish the peer review history of their article (what does this mean? ). If published, this will include your full peer review and any attached files.). If published, this will include your full peer review and any attached files.

If you choose “no”, your identity will remain anonymous but your review may still be made public.

**Do you want your identity to be public for this peer review?** For information about this choice, including consent withdrawal, please see our For information about this choice, including consent withdrawal, please see our Privacy Policy .-->.

Reviewer #1: No

**********

# Editor report: Acceptance letter

**DOI:** 10.1371/journal.pone.0345855.r006

**Authors:** José Antonio Ortega

PONE-D-25-08308R2

PLOS One

Dear Dr. Alamdo,

I'm pleased to inform you that your manuscript has been deemed suitable for publication in PLOS One. Congratulations! Your manuscript is now being handed over to our production team.

At this stage, our production department will prepare your paper for publication. This includes ensuring the following:

* All references, tables, and figures are properly cited

* All relevant supporting information is included in the manuscript submission,

* There are no issues that prevent the paper from being properly typeset

You will receive further instructions from the production team, including instructions on how to review your proof when it is ready. Please keep in mind that we are working through a large volume of accepted articles, so please give us a few days to review your paper and let you know the next and final steps.

Lastly, if your institution or institutions have a press office, please let them know about your upcoming paper now to help maximize its impact. If they'll be preparing press materials, please inform our press team within the next 48 hours. Your manuscript will remain under strict press embargo until 2 pm Eastern Time on the date of publication. For more information, please contact onepress@plos.org.

You will receive an invoice from PLOS for your publication fee after your manuscript has reached the completed accept phase. If you receive an email requesting payment before acceptance or for any other service, this may be a phishing scheme. Learn how to identify phishing emails and protect your accounts at https://explore.plos.org/phishing.

If we can help with anything else, please email us at customercare@plos.org.

Thank you for submitting your work to PLOS ONE and supporting open access.

Kind regards,

PLOS ONE Editorial Office Staff

on behalf of

Dr. José Antonio Ortega

Academic Editor

PLOS One
